# Seeing via Miniature Eye Movements: A Dynamic Hypothesis for Vision

**DOI:** 10.3389/fncom.2012.00089

**Published:** 2012-11-08

**Authors:** Ehud Ahissar, Amos Arieli

**Affiliations:** ^1^Department of Neurobiology, Weizmann Institute of ScienceRehovot, Israel

**Keywords:** active vision, fixational eye movements, neuronal phase-locked loop, temporal coding, neural coding, simple cells, thalamocortical loop, feedback

## Abstract

During natural viewing, the eyes are never still. Even during fixation, miniature movements of the eyes move the retinal image across tens of foveal photoreceptors. Most theories of vision implicitly assume that the visual system ignores these movements and somehow overcomes the resulting smearing. However, evidence has accumulated to indicate that fixational eye movements cannot be ignored by the visual system if fine spatial details are to be resolved. We argue that the only way the visual system can achieve its high resolution given its fixational movements is by seeing *via* these movements. Seeing via eye movements also eliminates the instability of the image, which would be induced by them otherwise. Here we present a hypothesis for vision, in which coarse details are spatially encoded in gaze-related coordinates, and fine spatial details are temporally encoded in relative retinal coordinates. The temporal encoding presented here achieves its highest resolution by encoding *along* the elongated axes of simple-cell receptive fields and not across these axes as suggested by spatial models of vision. According to our hypothesis, fine details of shape are encoded by inter-receptor temporal phases, texture by instantaneous intra-burst rates of individual receptors, and motion by inter-burst temporal frequencies. We further describe the ability of the visual system to readout the encoded information and recode it internally. We show how reading out of retinal signals can be facilitated by neuronal phase-locked loops (NPLLs), which lock to the retinal jitter; this locking enables recoding of motion information and temporal framing of shape and texture processing. A possible implementation of this locking-and-recoding process by specific thalamocortical loops is suggested. Overall it is suggested that high-acuity vision is based primarily on temporal mechanisms of the sort presented here and low-acuity vision is based primarily on spatial mechanisms.

## Introduction

During fixation, the eyes move across several arcminutes with amplitudes that fall off with the scanning frequency (Findlay, 1971; Eizenman et al., 1985). These fixational eye movements (FeyeM) cover the entire spectrum between ∼1 and more than 100 Hz, with increased power in two main frequency ranges: one between ∼1 and ∼20 Hz (“drifts”) with amplitudes of less than ten to a few tens of arcminutes (′) and another between ∼40 and ∼100 Hz (“tremor”) with amplitudes of a few arcseconds (″) to a few arcminutes (Ratliff and Riggs, 1950; Barlow, 1952; Yarbus, 1967; Bengi and Thomas, 1972; Shakhnovich, 1977; Coakley, 1983; Eizenman et al., 1985). Microsaccades (brief movements of a few to a few tens arcminutes) usually interrupt these movements a few times per second and move both eyes simultaneously in the same direction (Krauskopf et al., 1960; St Cyr and Fender, 1969; Moller et al., 2002; Engbert and Kliegl, 2004). Drifts and microsaccades usually counteract each other such that the eyes repeatedly scan the same fixational area (St Cyr and Fender, 1969; see reviews by Steinman and Levinson, 1990; Martinez-Conde et al., 2004).

Since time constants of retinal responses are in the order of 30–100 ms (Sakuranaga et al., 1987; Nirenberg and Meister, 1997; O’Brien et al., 2002), snapshots of spike activity of retinal ganglion cells, even if taken within a brief time interval, usually include spikes that were evoked by FeyeM occurring tens of ms before (Barlow, 1952). These slow retinal traces would smear the perceived image if the readout circuits would assume spatial coding alone, namely, that the spatial map of retinal spikes at a given moment provides all the information that is required for perceiving the external image accurately. However, smearing could be avoided if temporal delays between retinal spikes would be appropriately processed by the visual system (Ahissar and Arieli, 2001). Traditionally, temporal mechanisms had not been considered for the processing of stationary objects, whereas they were considered for the processing of moving objects, perhaps because stationary objects were intuitively conceived to induce stationary stimuli on the retina. Currently, however, it is widely accepted that temporal aspects both in the external world and in the encoding need not be averaged out when analyzing or modeling visual coding because, as far as the retina is concerned, stationary stimuli do not exist during natural viewing–retinal images are always moving (Kagan, 2012).

In fact, our vision of stationary images depends crucially on these movements. Virtually all retinal ganglion cells respond to transients of light, and many respond only to transients (Hartline, 1938; Hubel, 1988). The dependency of vision on transients is so strong that stabilization of a retinal image not only impairs visual performance (Kelly, 1979; Tulunay-Keesey and VerHoeve, 1987; Rucci and Desbordes, 2003; Rucci et al., 2007), but eventually causes the image to fade away (Fading time can be anything between 80 ms to a few seconds, depending on the stabilization method, Riggs et al., 1953; Pritchard, 1961; Yarbus, 1967; Ditchburn, 1973; Coppola and Purves, 1996). Thus, an evolutionary selection for FeyeM may have emerged to prevent retinal adaptation when viewing stationary objects. Whether FeyeM emerged first and retinal sensitivities adapted or vice verse is not known and is not relevant to the current discussion. Similarly, whether FeyeM result from uncontrolled muscular noise (Carpenter, 1988), or (/and) are centrally controlled (Shakhnovich, 1977; Coakley, 1983; Eizenman et al., 1985; Martinez-Conde et al., 2004) is not directly relevant to the current discussion, although it is certainly relevant to the understanding of vision in general. In this respect it is important to mention recent convincing evidence supporting internal control of FeyeM in a way that optimizes retinal processing of natural images (Kuang et al., 2012). In any case, what is directly relevant to the current discussion is the fact that the two phenomena are congruent: FeyeM continuously provide the transient stimuli required to activate retinal cells. This congruency constrains the possible ways in which retinal information can be encoded accurately. On the one hand, it precludes a pure spatial code at the retina, since the spatial image is smeared. On the other hand, it generates accurate temporal coding of the image, since retinal cells are predominantly activated *when* their receptive field (RF) crosses an edge of an image.

What are the relative contributions of spatial and temporal codes to retinal outputs? There is no doubt that spatial coding, in which spatial location of external stimuli is coded by ganglion cell identity and illumination intensity by the cell’s firing intensity, would work just fine for low resolution vision, where low resolution here means resolution coarser than the extent of FeyeM (around 10′). This is because integration of retinal responses over fields larger than the extent of FeyeM is hardly affected by FeyeM. The question is whether spatial coding would work for fine vision, i.e., for spatial details smaller than 10′, and specifically for hyperacuity vision, i.e., for spatial details smaller than 0.3′. It might be argued that the visual system is still using the spatial code for fine and hyperacuity vision, while applying some error-correction mechanisms to overcome the effect of FeyeM. It appears that error-correction mechanisms proposed in the past for the visual system, such as shifter circuits (Anderson and Van Essen, 1987) or interpolation circuits (Barlow, 1979; Crick et al., 1981), are not suitable for FeyeM (Ahissar and Arieli, 2001). Recently, an elegant mechanism that uses FeyeM statistics to correct for their effect in a discrimination task had been suggested (Pitkow et al., 2007). However, it is not yet clear whether mechanisms of this type can correct for FeyeM when the object is not known in advance or when more than one feature of the object are to be perceived or when multiple objects are present in a complex background such as in natural vision. But perhaps the strongest argument against the use of spatial-rate coding for fine vision is that the retinal rate code is an order of magnitude less accurate and reliable than the retinal temporal code (Berry et al., 1997). In every response, occurring upon an illumination transient, the timing of the first spike in a burst is accurate and reliable while the number and rate of following spikes is variable and not consistent (Reich et al., 1997). It takes a great deal of justification for the visual system to ignore the most informative code and use the less informative one when processing fine spatial details (Van Rullen and Thorpe, 2001; see A Simple Quantitative Account Against Spatial Retinal Coding of Fine Spatial Details in Appendix 2, and Meister and Berry, 1999).

We present here a mechanism for temporal encoding and decoding in the visual system. We consider it as a new dynamic theory of vision, to follow previous original suggestions by Marshal and Talbot (1942), Bryngdahl (1961), Arend (1973), Ahissar and Arieli (2001), Rucci (2008). The analysis presented here does not aim to disprove the possibility of spatial coding for fine details – this remains to be an experimental question. The analysis presented here is aimed at showing that in the visual system a temporal encoding-decoding scheme is possible, and to show a possible specific implementation; we do not claim that this is the only possible implementation, or that the system must implement dynamic processing this way. We do claim that the mechanism presented here is a plausible and efficient way to process retinal outputs and that it is consistent with a large body of data.

## A Relative Time Hypothesis for Vision

### Scope of the hypothesis

We refer here to the entire spectrum of FeyeM, excluding microsaccades, and to all two-dimensional visual cues that define visual objects (we term those 2D cues), excluding depth cues. Our analysis that follows will show that different 2D cues can be encoded by different variables of retinal responses: shape by inter-receptor temporal phases, texture by instantaneous intra-burst rates, and motion by inter-burst temporal frequencies. The information carried by these retinal signals is valid only during an individual cycle of eye movement, and thus must be read out during that cycle. We will show how an NPLL-like locking-and-decoding process, implemented by thalamocortical loops of the visual system, can set the timing for meaningful cortical processing of 2D cues and decode some of these cues. Decoding of relative depth information is postulated to be implemented by an independent mechanism, possibly a mechanism that relies on receptor activation during microsaccades, since these movements are predominantly in the horizontal direction (Liang et al., 2005) and are highly coordinated between the two eyes. Outlines for such an algorithm are described in Sampling of Relative Depth Information by Microsaccades in Appendix 2; the rest of the article deals with the processing of 2D details via non-saccadic FeyeM.

### Assumptions

1. Visual perception is assumed to be a process that builds on sensory data acquired during either fixation or fixational pauses, i.e., pauses between adjacent saccades (Barlow, 1952). During these periods, the eye rotates back and forth (FeyeM) and thus “scans” or “palpates” the image. Illumination changes induced during these movements activate retinal photoreceptors and dominate the input to the LGN.
2. Eye movements during fixation and fixational pauses typically consist of bursts of a few (2–4) cycles, and each burst has a dominant oscillatory frequency (Barlow, 1952; Byford and Stuart, 1961; Matin and Pearce, 1964; Bengi and Thomas, 1968; Coakley, 1983; Moller et al., 2002; Bosman et al., 2009). Data recorded from five subjects while fixating on single points or grating images, or while freely viewing a natural image, demonstrate this behavior (see Examples of Human FeyeM in Appendix 2).
3. Responses of visual neurons during natural viewing cannot be predicted from responses to flashed stimuli on paralyzed eyes. During natural vision there are no flashes. Rapid activations that are induced by saccades, and could potentially be compared to flashed stimuli, always involve retinal motion. Furthermore, downstream processing of the retinal signals evoked immediately after saccades likely involves significant suppression and distortion that are not present when flashing on passive eyes (Carpenter, 1988; Ross et al., 2001; Zirnsak et al., 2010; Hamker et al., 2011).
4. Retinal activations that are relevant for perception during natural viewing are generated by retinal motions caused by object motion or eye rotations (Ahissar and Arieli, 2001).
5. Functional anatomy exposed by flashed stimuli (Hubel, 1996; Ferster and Miller, 2000; Reid, 2001) is assumed to be valid also during natural viewing. During natural viewing, image scanning via FeyeM induces a variety of activation levels in overlapping RFs at various orientations; all these activations, of which the most intense ones have no priority, are assumed to be relevant for visual perception.
6. Temporal precision at the retina and LGN is in the order of 1 ms. Temporal precision at the retina was measured in salamanders and rabbits and was found to depend on stimulus contrast. The temporal jitter (for repeating identical stimuli) of fast OFF cells in the salamander retina is a power function of the stimulus contrast, with an exponent close to −0.5, such that at contrast of 35% the jitter is 4.4 ms (Berry et al., 1997); higher contrasts were not tested. In the cat’s LGN, with moderate contrast levels, many individual cells show time precision of <1 ms (Reinagel and Reid, 2000). Here we assume that the temporal jitter in the human retina is not larger than that of the salamander (whose retina can be compared to the human’s peripheral retina) or the cat, and that it continues to decrease with increasing contrasts. In our simulations of human foveal vision we assume temporal jitter ≤1 ms in the retina and LGN; the results, however, do not depend on the exact value of the temporal jitter.

### Format of the paper

The primary description of the hypothesis uses statements and derivations expressed in common terminology of experimental neuroscience, supported by schematic figures. The internal consistency of the hypothesis is demonstrated using formal definitions and derivations, which are presented in Appendix 1 and referenced in the relevant places in the text, and computer simulations. Data that support our assumptions are presented in Appendix 2.

### Sampling via simple receptive fields

2D spatial details are sampled by each eye independently. The information conveyed to the visual system depends on the external scene, the pattern of FeyeM of that eye, and the structure of afferent RFs. According to the afferent scheme suggested by Hubel and Wiesel (1962), which is consistent with a large body of data (reviewed in Hubel, 1996; Ferster and Miller, 2000; Reid, 2001), cortical simple cells receive inputs from elongated arrays of retinal ganglion cells via parallel channels through the LGN. These elongated arrays are composed of ganglion cells that share the same polarity, ON or OFF; interestingly, this segregation seems to be refined by FeyeM during development (Rucci et al., 2000). As a result of this anatomical arrangement, simple cells respond most vigorously to bars of the appropriate polarity that are oriented parallel to the elongated axis of their RF and are flashed on or moved across it (Hubel and Wiesel, 1962). However, they do not respond only to oriented bars. Both in cats and primates simple cells respond also to single dots flashed within their RF (Hubel and Wiesel, 1962; Gur and Snodderly, 1987; Hirsch et al., 1995; Tsao et al., 2003). Simple cells and other cortical cells respond vigorously to single spots or random dot patterns that are moved across their RFs in various directions (Hubel, 1958; Skottun et al., 1988; Pack et al., 2003a; Grunewald and Skoumbourdis, 2004). Interestingly, simple cells even show selectivity for dots moving *along* the elongated axis of their RFs (Geisler et al., 2001). As a result, when complex stimuli are moved across a stationary retina, simple cells respond in a variety of conditions, and are not limited to lines of a specific orientation (Creutzfeldt and Nothdurft, 1978). Scanning velocities in the above experiments were a few degrees per second, comparable with eye velocities during FeyeM (Riggs et al., 1954; Bengi and Thomas, 1968). Indeed, cortical neurons respond reliably also in the reverse, natural case, in which a moving eye scans stationary images during fixation (Snodderly et al., 2001). This latter study demonstrates clearly that the primate visual system tracks accurately the contrast changes scanned by the eye during FeyeM.

The size of simple RFs decreases as they get closer to the fovea. However, as recordings approach the fovea, measurements of RF size become difficult due to eye movements, especially in awake primates. Measurements done with the aid of image stabilization, i.e., while moving the image along the on-line-recorded trajectory of the FeyeM, show that at eccentricities of 2–9°, the width of subfields of simple RFs in layer 4 averages around 12 arcmin, and can be as low as 5 arcmin or less (Kagan et al., 2002). We are not aware of stabilized data from smaller eccentricities. Extrapolation of these data suggest that the width of subfields of foveal simple cells is expected to be at the level of a single-cone, which is consistent with a number of anatomical, physiological, and psychophysical indications (Polyak, 1941; Daniel and Whitteridge, 1961; Smallman et al., 1996; McMahon et al., 2000). It is not yet known whether foveal simple cells possess a single subfield (monocontrast cells Kagan et al., 2002) or more, and what aspect ratios characterize their subfields. In the following we will thus analyze the responses of single subfields of foveal simple cells, and will term such a subfield sRF. An ON sRF is a retinal field in which a given simple cell responds to transitions from dark to light, and an OFF sRF is a retinal field in which a given simple cell responds to transitions from light to dark. The examples depict arrays of foveal sRFs of a similar polarity, and a size of 1 × 4 cones. Whenever relative cortical coding is addressed, it relates to relative coding among sRFs of the same polarity.

### The feedforward signals: Coding along vs. across sRFs

**Figure 1:**
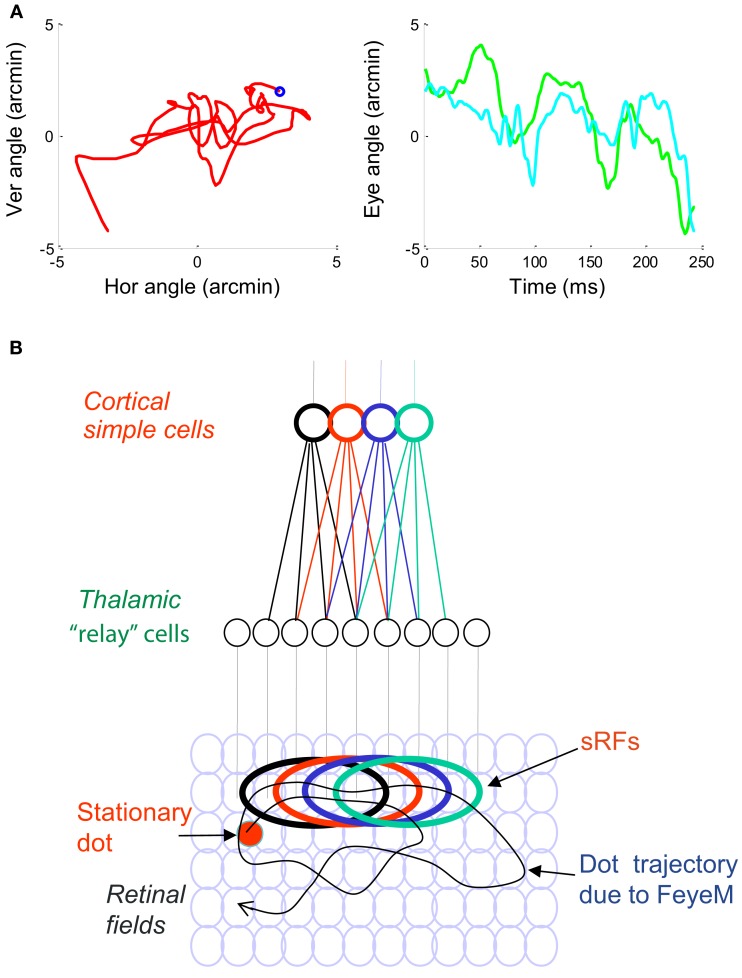
**(A)** A short epoch of FeyeM recorded from a human subject fixating on a cross (see Examples of Human FeyeM in Appendix 2 for methods and Figures A1 and A2 in Appendix 2 for more examples). Left, eye rotation trajectory on a 2D plane; coordinates (0, 0) denote cross center and the blue circle denotes eye angle at time = 0. Fight, horizontal (green) and vertical (cian) coordinates of eye angle as a function of time. FeyeM data courtesy of Dr. Moshe Fried. **(B)** A schematic description of a retinal trajectory of a stationary external dot (red) over a moving retinal mosaic of foveal ganglion RFs. Cortical simple cells receive their inputs, via thalamocortical neurons, from elongated retinal fields.

In the natural case, the entire visual field is sampled synchronously by all retinal cells at the frequency of the FeyeM, and each location in the visual field is sampled in succession by neighboring retinal cells (Figure 1). During fixation, a single external dot “draws” contours on the retina, which cross sRFs at various directions, determined by the direction of movement of the eye (Figure 1). If responses of cortical simple cells were based on feedforward (FF) circuits only (“FF response”), their duration would be determined by the duration of the intersection of the dot trajectory and the sRF. As we will see later, the actual responses of simple cells involve additional components contributed by the thalamocortical closed loop circuitry. However, in order to understand retinal encoding along and across sRFs, we virtually open these loops and analyze the FF responses as if no corticothalamic feedback is functioning.

**Figure 2:**
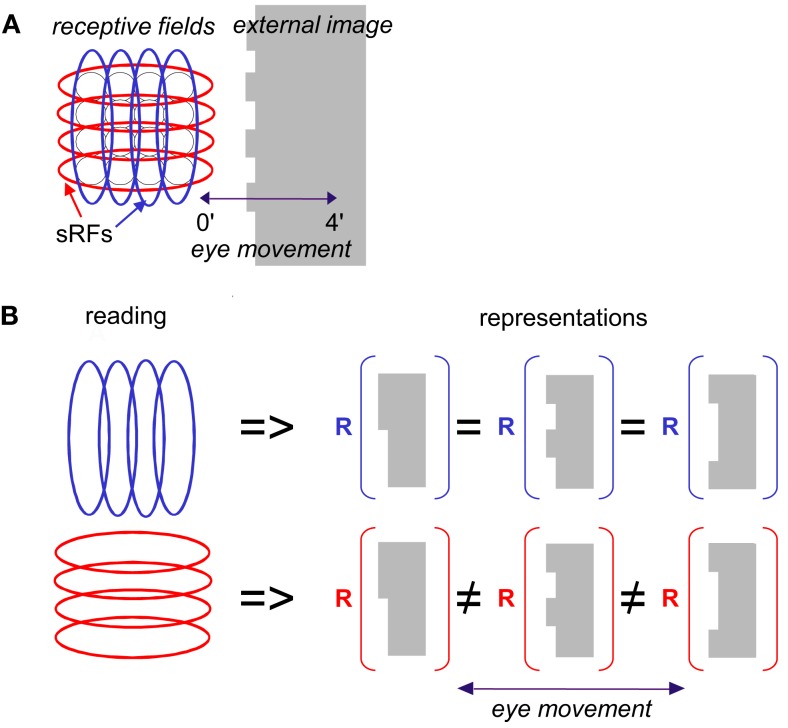
**Scanning of a stationary edge by drift**. **(A)** Retinal mosaic (as in Figure 1) scans a stationary object whose left edge is patterned during a horizontal drift. The peak-to-peak amplitude of the horizontal projection of FeyeM is 4′. Retinal ganglion RFs are indicated by black circles, and sRFs with horizontal (red) and vertical (blue) orientations are indicated by ellipses. **(B)** Scanning *along* the long axes of sRFs increases spatial resolution. In this example, reading sRFs oriented parallel to the global orientation of the patterned edge of an external image (blue) cannot generate different representations (*R*) for different edge patterns, whereas reading sRFs oriented perpendicular to the edge (red) can (using temporal coding).

Feedforward responses contain information about fine spatial relationships within the image. The resolution of this information depends on the orientation of the sRF with respect to the scanned image. Consider, for example, the schematic example in Figure 2A. When traversed by the eye, the external image is encoded by sRFs oriented in various directions, all of which provide useful information to the brain. Traditionally, processing of oriented edges, such as the patterned edge, had been assumed to rely on spike rate (or count) information which represents the matching between the orientations of the sRF and the edge. For example, the global orientation of the patterned edge in Figure 2A can be determined by the identity of maximally active sRFs – vertical ones in this case (blue ellipses). The orientation angle of the edge can be approximated with high resolution by interpolating firing intensities of simple cells having nearby orientations (Wilson, 1986). However, this kind of coding is limited in spatial resolution. Simple cells that are oriented parallel to edges would respond with the same mean intensity to edges whose general orientations are similar, regardless of their fine shape (e.g., Figure 2B). In other words, the sampling resolution of an array of sRFs is the lowest along an axis parallel to their orientation. Furthermore, the span and resolution of this intensity coding is limited by the maximal number of spikes that can be generated during a single scan of the edge. With foveal sRFs of one to two ganglion cells width, and FeyeM velocity of about 100 ′/s, the entire width of the sRF crosses the edge within about 5 ms, leaving time for one to two spikes per sRF. This poses significant constrains on the number and reliability of sRFs required for reaching perceptual resolution (see also A Simple Quantitative Account Against Spatial Retinal Coding of Fine Spatial Details in Appendix 2).

Another important factor that enormously constrains the way this information can be read is the existence of FeyeM. Simple cell responses depend crucially on the direction of eye movement. Thus, for example, if the movement of the eye is perpendicular to the orientation of the edge (e.g., Figure 2), and its velocity is appropriate, vertical sRFs will fire at maximal rate. However, response rate will decrease significantly when the direction of FeyeM becomes more parallel to that orientation. Moreover, movement in opposite directions would yield significantly different responses in most sRFs (see Snodderly et al., 2001). Thus, reading the intensity code of simple cells must take into account the direction of FeyeM at any given moment or employ a mechanism that somehow corrects for them (e.g., Anderson and Van Essen, 1987). To retain high resolution, such a mechanism must operate pre-cortically, which does not seem to be plausible (Ahissar and Arieli, 2001).

So how does the visual system restore the information that is lost by FeyeM smearing? The simple answer is that the visual system does not need to restore this information because it is not lost. This information is conveyed in a form of a temporal code that is encoded *along* the elongated axes of sRFs. In this paper we thus suggest an alternative to the classical encoding scheme, an alternative which we believe is consistent with existing data at least to the same degree that the classical one is. We suggest that information obtained *across* the elongated axes of sRFs (e.g., blue vertical sRFs in Figure 2) is used for coarse image analysis, while fine details are encoded *along* the elongated axes of the sRFs (Figure 2, red, horizontal sRFs). With this encoding scheme, spatial information is encoded by temporal neuronal variables, and hyperacuity resolution is an intrinsic property of the encoded signals (Ahissar and Arieli, 2001). As we will see below, this temporal encoding captures the fine differences between the different edges presented in Figure 2B.

### Temporal encoding

**Figure 3:**
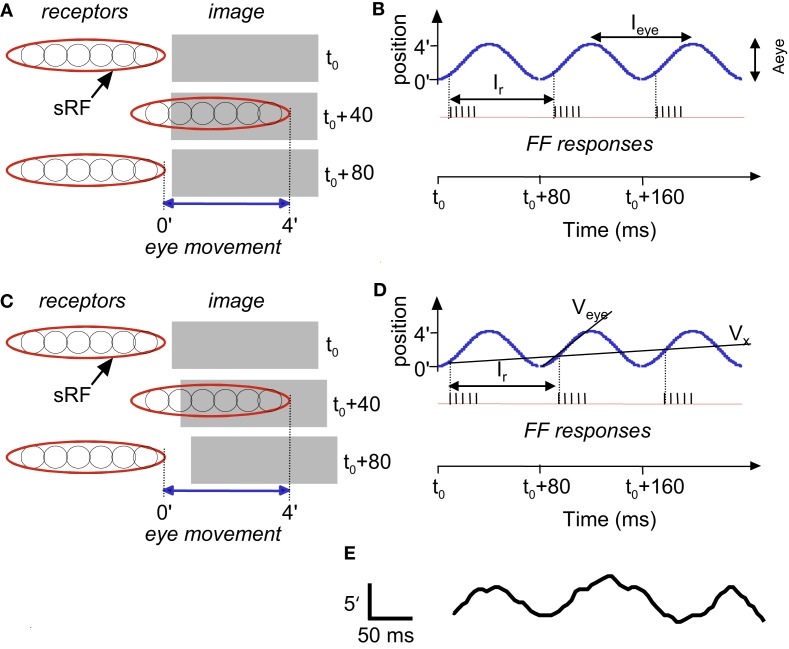
**Temporal encoding along the long axis of sRF during drift: a schematic description**. Encoding of stationary **(A,B)** and moving **(C,D)** images are depicted. A single horizontal sRF, shown in three time frames (*t*_0_, *t*_0_ +40 ms and *t*_0_ +80 ms), traverses a dark rectangle, which is longer than the peak-to-peak amplitude of the horizontal FeyeM. The horizontal FeyeM amplitude (peak-to-peak) is 4′ and the frequency is 12.5 Hz. **(A)** Left-most and right-most positions of the sRF relative to the position of a stationary image, within each single cycle of FeyeM. **(B)** Eye trajectory along the horizontal direction (blue curve) and neuronal responses (vertical lines) composing the FF signal of the sRF in **(A)**. *I*_eye_ and *A*_eye_ are the period (1/frequency) and amplitude of FeyeM, ***I****_r_* – is the inter-burst period of the FF response, and *t*_0_ is an arbitrary time. One spike is emitted per crossing of the contrast edge by each of the ganglion RFs. **(C)** Left-most and right-most positions of the sRF relative to the positions of a moving dark rectangle. **(D)** Eye and image trajectories along the horizontal direction and FF responses of the sRF in **(C)**. *V*_eye_ is the average eye velocity in the protraction direction, and *V_x_* is the image velocity. **(E)** An example of a human horizontal FeyeM oscillatory epoch, aligned with our schematic example for comparison (courtesy of Flemming Møller; see Figure 4 in Moller et al., 2002).

**Figure A2:**
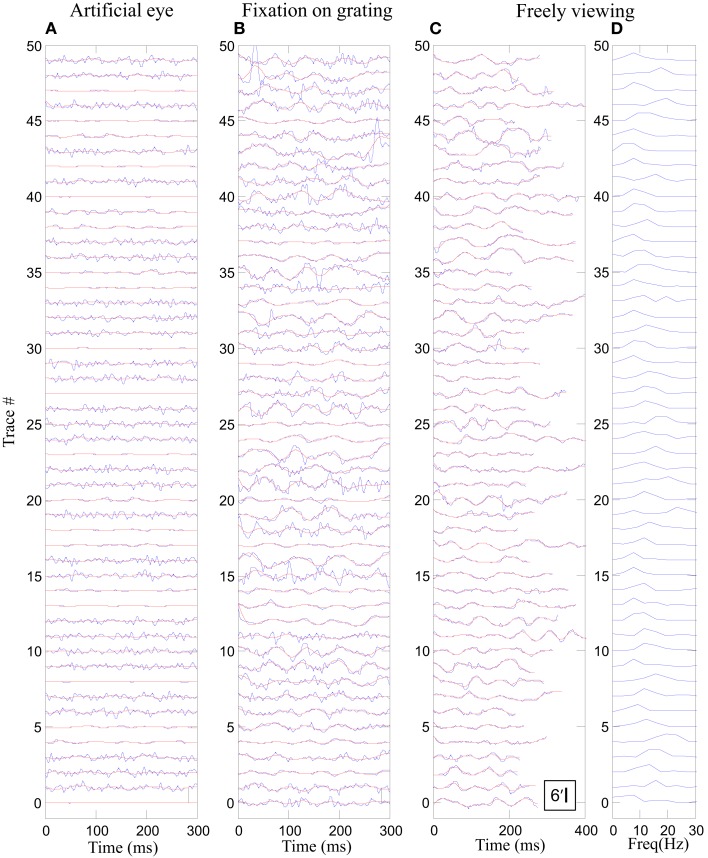
**FeyeM recorded from five human subjects while wearing masks with artificial eyes (A), fixating on a single spot surrounded by horizontal or vertical gratings (B), freely viewing a colorful image (C,D)**. Traces are depicted for arbitrary windows of 300 ms **(A,B)** or for fixational pauses – period starting 30 ms after a saccade and ending 10 ms before the next saccade. Raw traces are depicted in blue and smoothed traces (Butterworth, four poles LPF at 40 Hz) in red. Calibration bar **(C)** 6 arcmin. **(D)** Power spectra of the traces in **(C)**. Data are courtesy of Dr. Moshe Fried.

While the mechanisms of spike generation at the retina are not completely understood, it is known that they can involve integration times of tens of ms (Meister et al., 1994; Chichilnisky, 2001). Importantly, however, onset timing uncertainty in the retina and LGN is in the order of 1 ms (Berry et al., 1997; Reinagel and Reid, 2000). Here, thus, we assume that the temporal precision of retinal ganglion cells, and of relative delays between neighboring ganglion cells, are reliable at the level of 1 ms. Based on that, the entire set of spatial relationships in an image can be encoded by a set of temporal delays between neuronal events (Ahissar and Arieli, 2001). Figure 3 demonstrates, schematically, such precise temporal encoding for stationary (Figures 3A,B) and moving (Figures 3C,D) stimuli: a horizontal sRF (red ellipse), composed of six OFF-type retinal ganglion RFs, scans an image during three cycles of an oscillatory epoch of horizontal eye movement (Figure 3A). Epochs of two to four oscillatory cycles of a fairly constant frequency are abundant in human and monkey FeyeM data (Barlow, 1952; Byford and Stuart, 1961; Matin and Pearce, 1964; Bengi and Thomas, 1968; Coakley, 1983; Moller et al., 2002; Bosman et al., 2009); one such example is depicted in Figure 3E (see also Figure A2 in Appendix 2).

Figure 3B shows a burst of five spikes during the rightward movement, generated by the retinal ganglion cells that are incorporated within the sRF that scans the image. Note that with this horizontal eye movement the response is generated only during rightward movement, which occurs because this sRF is composed of OFF-type fields. With ON-type fields, responses would have been generated only during leftward movement. In the following, we refer to the direction of FeyeM that generates responses in a particular sRF as its “protraction direction,” whereas the opposite direction is referred to as “retraction direction.”

#### Encoding of motion

Movements in the visual field produce Doppler-shifts effect of the retinal activation frequencies. For example, a light-to-dark edge that is moving away from an OFF-type sRF (Figure 3C) lengthens the intervals between successive retinal activations that are caused by the FeyeM oscillation (Figure 3D; compare the phases of burst onsets in the FeyeM cycle). This lengthening result in decreased inter-burst and intra-burst frequencies, and the decreases are in proportion to the velocity of the object (*V_x_*). Movement in the opposite direction will increase these frequencies (Retinal Frequencies and Encoding of Motion in Appendix 1, Eq. 3). These Doppler-shifts represent the component of velocity that parallels the trajectory of the eye. Movement components perpendicular to the trajectory of the eye might be detected by other mechanisms, possibly non-foveal ones. For example, ganglion cells of salamander and rabbit retina detect relative motion induced by FeyeM-like movements (e.g., Olveczky et al., 2003). Their detection mechanism probably relies on integration of many photoreceptors by each ganglion cell, an integration that does not occur in the human fovea (Polyak, 1941). Such retinal mechanisms, thus, could serve peripheral motion detection.

#### Encoding of shape

**Figure 4:**
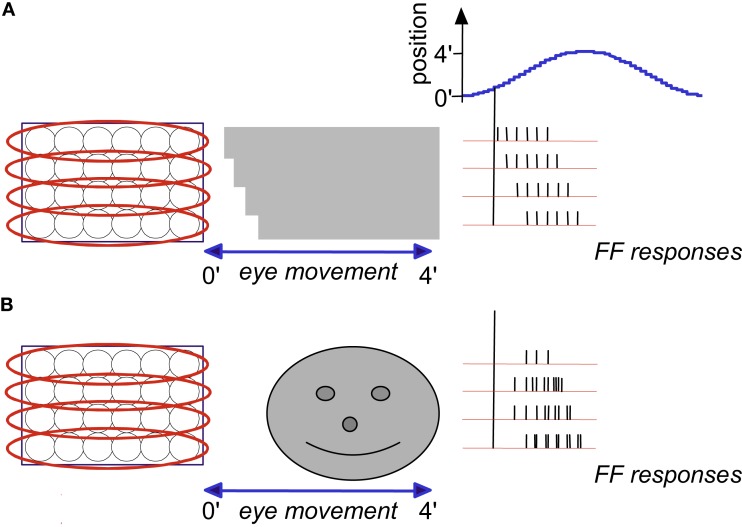
**Temporal encoding of texture during drift: a schematic description**. Retinal mosaic scans a stationary smooth **(A)** and patterned **(B)** surfaces during horizontal drift (movement parameters and other conventions as in Figures 2 and 3). Every crossing of a light-to-dark luminance border by a single ganglion RF generates a single spike. For clarity, spikes are drawn only during one direction of drift (the “protraction” direction).

Encoding of shape, i.e., of the outline of an object, is demonstrated by the scanning of a stationary terraced edge (Figure 4A). Since the retinal movement of the stationary object is spatially coherent, the relative temporal phases of the bursts, across the corresponding sRFs, encode the relative spatial locations of the terraces (Temporal Encoding in Appendix 1, Eq. 1). In this example, a single cycle of FeyeM (4′ at 12.5 Hz) is shown. A burst of six spikes per FeyeM cycle occurs as the six horizontal OFF-type sRFs traverse the objects. Spatial offsets of 15″ are encoded by temporal delays of ∼2.5 ms (*V*_eye_> = 4′/40 ms). Note that with encoding along the elongated axes of sRFs, neither the firing rate nor the total spike count provide information about the relative locations of the edges in the external image.

#### Encoding of texture

Encoding of texture, i.e., of the characteristics of surfaces such as patterning and spatial frequencies, is demonstrated by the scanning of two gratings (Figure 4). Here, the outline of the object (its shape) is again encoded by the relative onset phases of the FF bursts. However, unlike the case of a smooth surface (Figure 4A), a patterned surface induces additional spikes in each ganglion cell: each ganglion cell fires every time it crosses a luminance change while scanning the surface (Figure 4B). The mean spatial frequency of the surface is encoded by the mean firing rate during protraction (compare Figures 4A,B). The exact pattern is encoded by the temporal phases. In the following we will assume smooth surfaces for simplicity.

### Encoding of FeyeM direction and resolving image details by sRF structure: 1D vision

Most of sRFs contain a “rod” of one polarity (ON or OFF) surrounded by flanks of the opposite polarity (Hubel and Wiesel, 1962). This special structure produces a kind of an isolated corridor through which image details are encoded in only one direction – along the elongated axis of the sRF. With typical FeyeM velocities, an image contrast scanned by a sRF in a direction not parallel to its elongated axis will not excite the central “rod” due to the inhibition activated at the flank a few ms before. This protected corridor offers two important functions. First, it allows independent processing of different axes of the visual image, e.g., vertical vs. horizontal (1D vision). Second, it provides information about the direction of the FeyeM. For example, when scanning velocity is in the working range of the flank-center inhibition a vertical FeyeM will not activate a horizontal sRF and a horizontal FeyeM will not activate a vertical sRF, whatever image is scanned. Note also that complex cells are even better encoders of FeyeM direction (and perhaps also speed) due to their integration of several SCs of the same orientation. Moreover, integrated activity of different horizontal complex cells can signal onset times of cycles of FeyeM in the horizontal direction, and that of vertical complex cells in the vertical direction.

### Temporal decoding

Decoding of temporally encoded signals can be implemented by various neuronal algorithms (Carr, 1993; Mauk and Buonomano, 2004; Theunissen et al., 2004). Specifically, encoding by a single common oscillatory modulation, as is induced here by FeyeM, can be decoded efficiently by simple FF neural networks (Hopfield, 2004). The efficiency of several of these decoding algorithms had been demonstrated for sensory systems other than the visual system. While some of these algorithms could also be plausible for the visual system, we prefer to suggest a specific algorithm which is based on the circuitry and cell physiology found in the primary visual thalamocortical system.

**Figure 5:**
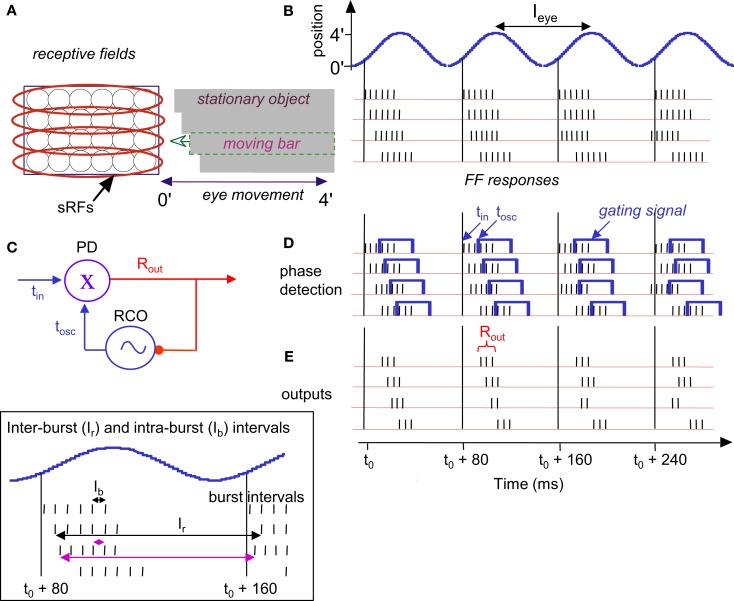
**Temporal encoding and decoding by the visual system**. **(A)** A retinal mosaic (as in Figure 2) upon horizontal FeyeM scanning of a stationary object and a moving bar, both of which have the same color and luminance (the moving bar is indicated by a broken rectangle). The bar starts to move leftward around *t*_0_ + 20 ms, at a velocity of 3 ’/s. The horizontal arrows mark the horizontal movement of the eye. The horizontal FeyeM amplitude (peak-to-peak) is 4′ and the frequency is 12.5 Hz. **(B)** FF responses of the horizontally oriented sRFs [red ellipses in **(A)**]. The sRF that scans the moving bar produces a FF response with higher inter-burst and intra-burst frequencies than its neighbors. **(C)** NPLL Decoding algorithm. PD, phase detector; *X*, phase detection, RCO, rate-controlled oscillator; ∼, local oscillations *t_in_*, input time; *t*_OSC_, oscillator time; *R*_out_, PD’s output representing the phase difference between *t_in_*, and *t*_OSC._ Blue, temporal signals; red, intensity (rate) signal. **(D)**. Implementation of phase comparison by gating. At each cycle, only those retinal spikes that arrive after the onset of the cortical feedback (blue squares) will “pass the gate.” Phase comparison is obtained by longer (shorter) delays yielding fewer (more) spikes due to decreased (increased) overlap between the inputs. Note the cycle-to-cycle temporal dynamics of both the retinal input and the periodic gating while processing the moving bar. Onset of the retinal signal is represented by *t_in_* in **(C)** and onset of the gating signal by *t*_OSC_ in **(C)**. **(E)** Output code of the proposed thalamocortical decoder. The stationary shape is represented by the temporal phase relationships among the outputs of the simple cells (first cycle). The velocity of the bar is represented by a change in the spike count (two instead of three spikes/cycle) of the simple cells [represented by *R*_out_ in **(C)**]. (Inset) Expansion of the second cycle in **(B)** to show inter-burst (*I_r_*) and intra-beurst (*I_b_*) intervals for stationary (black) and moving (magenta) bars.

For the description of the proposed decoding process, we depict an example similar to that of Figure 4A, but with the addition of a moving object (Figure 5A). Three of the horizontal bars that compose the external image are stationary and one (dashed rectangle) is moving leftward (Figure 5A). As a result, the FF frequencies of the sRF scanning the moving bar increase (Figure 5B and inset): the inter-burst period is shortened by ∼2.5 ms in each cycle, and the intra-burst period is shortened by a similar ratio. These changes are specific to the sRF scanning the moving bar; inter- and intra-burst periods remain unchanged for the FF signals that represent the stationary parts of the external image.

**Figure 6:**
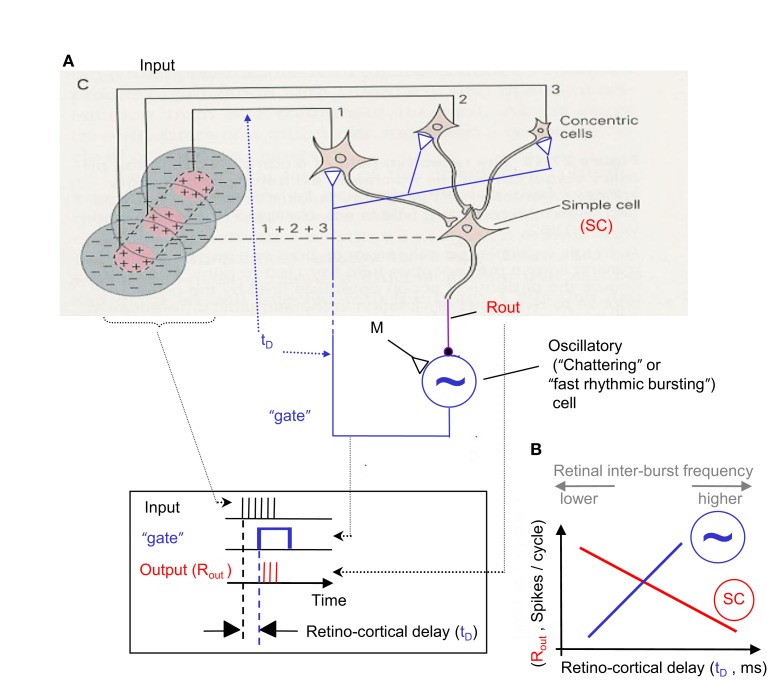
**NPLL implemented by a visual thalamocortical loop**. **(A)** A schematic description of the proposed thalamocortical closed loop decoder. The scheme is based on the schematic description of the FF connectivity suggested by Hubel and Wiesel (1962); the feedback connectivity added in blue closes the loop in a way that permits a PLL-like operation. Excitatory and inhibitory connections are represented by open triangles and solid circles, respectively. Dashed line indicates possible poly synaptic link. Input, retinothalamic input; SC, simple cells; M, modulatory excitatory input; ∼, oscillatory (“chattering”) neurons. Inset: implementation of the phase detection function by corticothalamic gating: the output is active only when both the Input and the “gate” are active. **(B)** Schematic phase plane of the two basic transfer functions of the loop. SC’s transfer function (red): output spike count (*R*_out_) decreases as the retino-cortical delay (*t_D_*) increases. Oscillatory cells transfer function (blue, dashed): *t_D_* increases as *R*_out_ increases (note reversal of axes here). The crossing point of the transfer functions is the set point for a specific retinal temporal frequency. The inter-burst frequency of the retinal input is directly related to *t_D_* and inversely related to *R*_out_.

Thus, shape and velocity are encoded at the retina by the temporal phases and frequencies of retinal bursts, respectively. Texture, as shown above, is encoded by the intra-burst firing pattern. The temporal structure of retinal activities is extremely reliable (Berry et al., 1997) and is well preserved up to the thalamus (Levick et al., 1972; Lee et al., 1981; Reinagel and Reid, 2000), and thus can be efficiently decoded by thalamic or thalamocortical circuits. In the following text and Figures 5 and 6 we demonstrate that decoding by thalamocortical circuits is possible.

#### Possible decoding by thalamocortical phase-locked loops

A constraint of the FeyeM-driven encoding scheme is that decoding must be locked to movements of the eye. Comparison of onset times or temporal periods of neighboring retinal cells is meaningless if done across different cycles of FeyeM, because eye position and velocity are not constant across cycles. Moreover, decoding of shape and velocity requires an accurate comparison, in each FeyeM cycle, of the burst onset times of retinal sRFs. These constrains suggest that the brain should either use efferent copies of the signals that control the FeyeM (Shakhnovich, 1977; Coakley, 1983; Eizenman et al., 1985), if they are available, or employ a mechanism that can lock to the afferent sensory signals; a combination of both strategies is also possible. Here we show how the brain could use a mechanism that locks to the sensory signals without the need for an efference copy.

Existing data from the mammalian visual system indicate that the visual cortex employs oscillatory mechanisms that can be used by the visual system to decode the information encoded by FeyeM (see *Cortical Oscillations* below). The most efficient oscillation-based temporal decoding mechanism is probably the phase-locked loop (Gardner, 1979), whose neuronal implementation is termed the neuronal phase-locked loop (NPLL, Ahissar, 1998). In its basic form a single NPLL consists of a phase detector (PD), whose output firing rate reflects the temporal phase difference between its inputs, and a rate-controlled oscillator (RCO), whose oscillating frequency is controlled by its input firing rate; the circuit forms a negative closed loop (Figure 5C). Experimental evidence suggests that thalamocortical NPLLs are involved in temporal decoding in the mammalian somatosensory system (Ahissar and Vaadia, 1990; Ahissar et al., 1997, 2000; Kleinfeld et al., 1999, 2002; Ahissar and Zacksenhouse, 2001; Sosnik et al., 2001; Moore, 2004; Zacksenhouse and Ahissar, 2006; Fox, 2008). We thus explore how could visual NPLLs, if exist, facilitate the readout of the information encoded by FeyeM.

Below (text and Figures 5 and 6), we describe how a single NPLL circuit, when implemented within a visual thalamocortical loop, could decode retinal information that is temporally encoded by FeyeM. We suggest that many such NPLLs are implemented in parallel, each having a slightly different intrinsic frequency, which together would cover the entire spectrum of FeyeM frequencies (see The Visual Motor-Sensory Loop and Figure 9 below and A Global View on the Perceptual Process in the Discussion; Ahissar, 1998). We suggest an implementation that relies on the same experimental data often providing the basis for purely spatial models. The suggested implementation, however, involves new functional interpretations of classical terms, such as orientation selectivity and optimal response. For example, one important deviation from classical convention is that optimal responses do not necessarily involve maximally possible firing rates, and computations are not necessarily based on feature detection signaled by firing rates.

#### Decoding algorithm

The core idea behind oscillation-based decoding is that the oscillations establish a local predictor for input timing (Figure 5C). By comparing the predicted time (*t*_osc_) with the actual time of the input (*t*_in_), a difference signal is generated (*R*_out_). In closed loop oscillation-based decoding, such as in the NPLL, the difference signal modulates the local oscillator in a negative feedback manner, namely it drives it in a direction that will approach a constant difference. If parameters are tuned correctly this negative feedback forces the local oscillations to track the timing of the input (see Loop Operation in Appendix 1 and Gardner, 1979). This generates a delayed internal replica of input times, which is locked to the FeyeM (Figure 5D). The local oscillations, being the best internal predictors for input timing, provide an internal marker for downstream processing, or a kind of a processing clock. In addition, when the loop is locked, the difference signal, *R*_out_, represents the deviations of the input timing from its own history (i.e., the input’s temporal modulations).

#### Thalamocortical implementation

A simplified model for a thalamocortical NPLL, which includes the minimal number of basic components and the constrains of known thalamocortical circuitry (White, 1989; Douglas and Martin, 1991; Lund and Yoshioka, 1991; Sherman and Guillery, 1996), is depicted in Figure 6. Thalamocortical projections converge on simple cells (SC, Hubel, 1996) in layer IV. These simple cells can be inhibitory, as in Figure 6, or could drive inhibitory interneurons (INH, Anderson et al., 1994). The inhibitory output project onto oscillatory neurons (such as “chattering” or “fast rhythmic bursting cells”; Gray and McCormick, 1996; Cardin et al., 2005; see also Ahissar and Vaadia, 1990; Llinas et al., 1991; Silva et al., 1991), which then close the loop by projecting onto the same thalamic cells that drive the loop’s simple cells. The corticothalamic projection might be implemented directly by oscillatory neurons in layer 6 (Cardin et al., 2005) or mediated by other, corticothalamic neurons, if chattering cells of layers 2/3 (Gray and McCormick, 1996) or cortical interneurons (Cardin et al., 2009) function as the local oscillators in specific circuits. In principle, the only component in the loop whose implementation requires more than a single neuron is the thalamic array. However, it is postulated that the entire thalamocortical loop has a “width” of more than one neuron, i.e., every component is implemented by a group of coupled neurons. Such an implementation is both more realistic and more efficient (Ahissar, 1998).

#### Decoding requirements

In order to obtain temporal decoding, the following three basic principles should be obeyed by a single loop (see Gardner, 1979): (i) the output of the simple cells (*R*_out_) should decrease as the retino-cortical delay (*t_D_*) increases (Figure 6B, red curve) and (ii) the period (inter-burst interval) of the oscillatory cells should increase as the output of the simple cells increases (Figure 6B, blue dashed curve); this change increases the delay between the retinal output and the cortical oscillatory cells (*t_D_*; note that for this function the ordinate describes the input, *R*_out_, and the abscissa describes the output, *t_D_*). The multiplication of the gains (slopes) of the two transfer functions described in (i) and (ii), the so-called open-loop gain (*G*), should thus be negative. In Figure 5D, an optimal set of loop parameters (*G* = −1) was depicted, such that the first correction of the oscillating period is the required one. When these requirements are obeyed, the thalamocortical loop is forced to “lock-in” to the retinal rhythm and to track its fluctuations (for constrains on locking dynamics, see Ahissar, 1998). The range of retinal frequencies for which these requirements are met by a specific NPLL is called the working range of that NPLL. The phase-locking machinery of NPLLs ensures that the processing is done cycle-by-cycle, with every processing cycle being matched to a specific FeyeM cycle. When an NPLL is locked to the FeyeM, outputs of its cortical simple cells recode the retinal frequency by spike count (per cycle), and preserve the temporal phase relationships between different retinal outputs (see *Cortical Representations* below). Furthermore, when an NPLL is locked to the FeyeM, the retino-cortical delay will be directly related to the inter-burst frequency of the retinal output (Figure 6B) both within each cycle (instantaneous increased retinal frequency will advance the next retinal spike while the next cortical oscillatory spike will maintain its previously determined prediction) and across cycles (due to the negative feedback nature of the loop which will enforce less inhibition on the cortical oscillatory cells and thus reduced *R*_out_ and thus increased *t_D_*; Ahissar, 1998).

As long as a neuronal loop obeys the requirements mentioned above, its exact implementation is not important, and could vary among different species or even among different individuals. For example, the circuit can be simple in cases where simple cells are by themselves inhibitory or when the oscillatory neurons project directly to the thalamus (as in Figure 6; Cardin et al., 2005), or it could be more complex if additional neurons relay information between the basic components, for example, between thalamocortical neurons and simple cells (Blasdel and Fitzpatrick, 1984).

#### Phase locking

In our model, the cortical oscillations produce internal expectations with regard to the timing of the next retinal output. These temporal expectations are manifested by the output of the oscillatory neurons, and thus also by the onset time of the corticothalamic gating signal (blue rectangles in Figure 6 inset and in Figure 5D). For every given retinal location, and its corresponding thalamocortical loop, deviations of retinal output from its expected timing are detected by thalamic “relay” neurons (i.e., PDs), affect the spike count of simple cells, and thereby, re-adjust the period of the subsequent cortical oscillation cycle. For example, assume that the input period changes from 80 to 77.5 ms, as is the case with the moving bar in Figures 5A,B. Since the cortical expectation for the second cycle in Figure 5 is still 80 ms, the phase difference between the input (Figure 5D, black spikes) and the cortical oscillations (blue rectangles) will increase (from 7.5 to 10 ms; second cycle in Figure 5D). Consequently the output of the simple cell(s) will decrease (from three to two spikes; Figure 5E), inhibition of oscillatory neurons will decrease, and therefore, the cortical oscillating period of the next cycle will decrease, thus tracking the change occurring at the input. In this example, optimal loop parameters (*G* = −1) were depicted such that the first correction of the oscillating period is the required one. Thus the loop is already stabilized at its new working point, one cycle after the change in the input occurred (i.e., at period = 77.5 ms, delay = 10 ms, output = 2 spike/cycle).

With less optimal parameters, complete stabilization might require more than a single cycle (Ahissar, 1998). In any case, if the retinal frequency is within the working range of the loop, the cortical period is forced to eventually converge to the retinal period, since (due to the negative feedback) the thalamic “error signal” will always drive the cortical oscillations in the direction that will tend to cancel the “error.” Note that the stabilized output spike count is determined by the inter-burst frequency; for decreasing retinal periods (increasing frequencies), the loop working point (Figure 6B) will move to the right, toward longer retino-cortical delays. Thus, when the frequency of FeyeM is continuously changing, as is the case with natural viewing, cortical oscillatory frequency will track those changes with a lag of at least one FeyeM cycle, where in each cycle the tracking error is reported by the output of simple cells. The emergence of phase-locking from the transfer functions of the loop is described in the following sections.

#### Cortical oscillations

Cortical oscillations can track input frequencies if their frequencies (i) are in the range of the input frequencies, and (ii) can be modulated by local cortical inputs (Cortical Oscillations in Appendix 1, Eq. 4). Both requirements are fulfilled by mammalian cortical oscillations. The spectral densities of human FeyeM and neuronal oscillations in the monkey visual cortex (Eckhorn, 1994) exhibit a striking similarity, both emphasize alpha and gamma modes (see Ahissar and Arieli, 2001). Oscillations at frequency ranges that match those of FeyeM had been observed in the visual cortex of cats, ferrets, and monkeys (Gray et al., 1989; Eckhorn, 1994; Gray and McCormick, 1996; Brumberg et al., 2000; Cardin et al., 2005; Bosman et al., 2009). The frequencies of visual cortical oscillations can be controlled locally (Gray and McCormick, 1996; Brumberg et al., 2000; Cardin et al., 2005) and can be modulated by and locked to external stimuli (Eckhorn et al., 1993; Gray and Viana Di Prisco, 1997; Cardin et al., 2005; Bosman et al., 2009), as is the case for other modalities (for review, see Ahissar, 1998).

Besides frequency ranges, the main difference observed between visual and other cortical oscillations is that visual oscillations have not been observed so far in the absence of visual stimuli. This might indicate that expression of cortical oscillations (e.g., translation of sub-threshold oscillations to spike activity) requires an additional excitatory input (Gray and McCormick, 1996; Cardin et al., 2005). Such an input (“M” in Figure 6) can be provided by an internal preparatory signal or an afferent stimulus-driven signal, and be shaped during development and by learning (Ahissar et al., 1998).

#### Thalamic phase detection

Thalamocortical neurons of NPLL are required to generate an output whose spike count decreases as the retino-cortical delay increases. This function is easily implemented by corticothalamic gating (Figure 6), in which thalamic transfer strongly depends on the cortical signal (Diamond et al., 1992; McCormick and Bal, 1994; Sherman and Guillery, 1996). In this gating mode, thalamic neurons fire only, or mostly, when retinal and cortical inputs overlap. When thalamic neurons are in this mode, and their cortical input is periodic, the result is periodic gating. At each oscillation cycle, thalamic output would be maximal when the two inputs fully overlap, and will gradually decrease as the delay increases until no overlap occurs (Thalamic Phase Detection in Appendix 1, Eq. 5). Thus, a computation of the phase difference between retinal and cortical activities and its recoding by spike counts occurs at each oscillation cycle. Operation of the PD is described schematically in Figures 5 and 6, in which the FF and feedback signals are illustrated as a spike burst and a rectangular function, respectively. The activity symbols in Figures 5 and 6 (FF-spike burst; feedback-a rectangular function) are used for illustrative purposes only; both signals are conveyed by discrete spikes, which in turn induce continuous post-synaptic potentials in their post-synaptic PD cells. The mechanism of thalamic phase detection resembles that of coincidence detection suggested for the auditory brainstem (Carr and Konishi, 1990), but with a wider response range; the response of thalamic phase detection decreases gradually as the phase difference increases while that of coincidence detectors decreases sharply. The gradual response profile is essential for the operation of the NPLL (see *Phase Locking* above).

#### Loop operation

In each oscillation cycle, loop variables depend on the input and on their values during the previous cycle (Appendix 1, Eqs 6 and 7). Thus, for any given constant input, the loop variables, such as simple-cell spike count and oscillator phase, will continue changing until frequency locking will be achieved. For each input frequency, the loop will stabilize on a different set point, i.e., different set of variable values (Appendix 1, Eqs 8–10). When the frequency of the input is not constant, cortical oscillations will track the changes in the input periodicity, aiming at (though never achieving) a new set point upon each change (Appendix 1, Eq. 11).

The dynamics of the loop following changes in FeyeM frequency are similar to those occurring following external motion (Figure 5, see *Phase Locking* above). Upon increased FeyeM frequency, the FF signals (of all NPLLs) arrive earlier than expected, while the gating signals appear at the time corresponding to the on-going expectation. The result is a decreased overlap in the thalamus (e.g., Figure 5D third row), thus decreased SC outputs, decreased inhibition on the oscillatory neurons, and thus increased oscillatory frequency. A similar process, with opposite signs, occurs with decreased FeyeM frequency.

Thalamic gating is not operational following sustained silent periods (McCormick and Bal, 1994; Sherman and Guillery, 1996). Therefore, when stimulating paralyzed eyes at low frequencies (<2 Hz), thalamocortical neurons would often be activated by retinal outputs even in the absence of cortical activity. If a burst of stimuli follows the first stimulus, a locking-in process is expected to be initiated in the corresponding NPLLs (see Figure 9 in Sosnik et al., 2001).

#### Cortical representations

Retinal periodicities are translated into latencies and spike counts of cortical simple cells (Figure 6B). Since the external velocity (*V_x_*) is encoded at the retina by a frequency shift, it is represented in the cortex by a decrement (Figure 5E) or increment in the number of spikes per cycle from the corresponding output of simple cells. Note that the coding of retinal velocity is differential: local motion is coded by the *difference* between the spike counts of neighboring simple cells. Thus, although absolute values of spike counts can vary with variations in FeyeM, the local difference due to external local motion will remain. In fact, differential coding applies to FeyeM-induced temporal coding in general. Image shape and texture, as already mentioned, are also coded differentially in this scheme, and are represented by temporal phase relationships among neighboring simple cells (shape, Figure 5E, first cycle; Cortical Representations in Appendix 1), and by relative intensities of intra-burst firing (texture; not shown).

### Processing of NPLL outputs by complex cells

Central processing of 2D details, as that of depth cues, is often described in terms of spatiotemporal filtering (Barlow, 1979; Fahle and Poggio, 1981; Burr et al., 1986; DeAngelis et al., 1993; Morgan and Castet, 1995). This description does not specify the actual processing carried by central circuits but indicates that the processing should be carried over both dimensions interactively. The NPLL circuits suggested here can be described as adaptive temporal filters, whose center frequency follows that of FeyeM. The outputs of these NPLLs, i.e., the outputs of cortical simple cells, are processed by complex cells across space. Adjacent simple cells feed the decoded temporal information (now in spike count code) to individual complex cells. Complex cells process (usually integrate) these inputs across the spatial dimension. The exact computation performed by complex cells, and their feedback interactions with thalamocortical NPLLs, are not yet clear. In any case, according to the scheme presented here, thalamocortical NPLLs are responsible for the temporal component, whereas cortical complex cells, interneurons, and the associated circuitry are part of the spatial component, of the implied spatiotemporal central processors of visual inputs.

### Phase encoding and recoding with natural FeyeM

**Figure 7:**
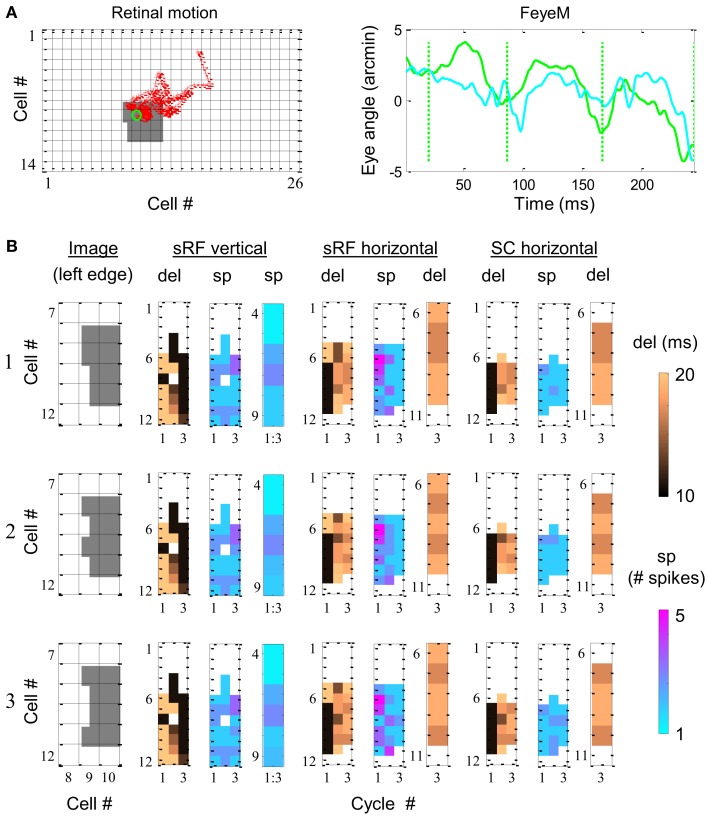
**Simulation of phase encoding and recoding of a stationary image with human FeyeM**. **(A)** FeyeM recorded from a human subject fixating on a cross (same example as in Figure 1A) were used to shift a stationary image over a retinal array of 14 × 26 OFF photoreceptors (left panel, retinal movement trajectory plotted in red; starting point circled in green). FeyeM cycle onsets were defined as the minima of a low-pass (cutoff frequency: 40 Hz) version of the horizontal trace (right panel, dotted vertical green lines). Size of all retinal RFs was set arbitrarily to 2′. **(B)** Dynamics of delay (del) and spike count (sp) across the three cycles of the FeyeM epoch in three columns of cells (vertical sRF, horizontal sRF and horizontal SC) and for three external images [rows 1–3, left edges of the images are depicted in the left panel of each row, the rest of the image was identical to the one in **(A)]**. Sp responses in vertical sRFs (mean over the three cycles) and del responses at the third cycle of sRFs and SCs are enlarged for the relevant cells. FeyeM data courtesy of Dr. Moshe Fried.

Although typically cyclic, FeyeM are not purely periodic. Trajectories of FeyeM are strongly modulated in both amplitude and frequency (see Examples of Human FeyeM in Appendix 2). Since, the induced eye movement is the same for the entire retina, the nature of relative time coding, i.e., coding spatial offsets by relative temporal delays across neighboring cells, should not depend crucially on the exact pattern of FeyeM. We tested this prediction using computer simulations. Three gray images with patterned left edges (Figure 7B, images 1–3) were scanned by a small retinal field that was moved according to the FeyeM recorded in a human subject (Figure 7A and Examples of Human FeyeM in Appendix 2). Ganglion cell firings were determined at a resolution of 1 ms (Meister and Berry, 1999; Reinagel and Reid, 2000) using a simple threshold crossing mechanism; note that an addition of integration-based mechanisms, while not expected to affect timing reliability and thus the relative temporal code, are expected to affect the absolute firing times of ganglion cells. The FF signals were conveyed by OFF-centered subfields of foveal simple cells (sRFs) of 4 × 1 (horizontal) and 1 × 4 (vertical) receptors, 1 arcmin each, flanked by symmetric inhibitory fields (1 receptor wide, Figure 2D in Hubel and Wiesel, 1962). Each sRF fed a “thalamocortical” loop that contained a RCO and SC and obeyed the NPLL equations (Eqs 4 and 5 in Appendix 1).

The dynamics of the simulated responses were analyzed in relation to FeyeM cycles. In each cycle, delay to the first spike (del) and total spike count (sp) were calculated. The vertical sRFs were blind to the fine offsets of the image’s left edge. The delays and spike counts conveyed by such sRFs (Figure 7B depicts one column of vertical sRFs) were almost identical for the three images. In contrast, horizontal sRFs exhibited different dynamics for each edge, resulting in a unique temporal representation during the 3rd cycle. The images were represented by the relative firing times of four horizontal sRFs (sRF #7–10 in Figure 7B, enlarged in a separate panel): for image 1, sRFs 7 and 8 exhibited relatively short delays from cycle onset while sRFs #9 and 10 exhibited relatively long delays (short-short-long-long). In a similar manner, images 2 and 3 were represented by short-long-short-long, and short-long-long-short delay patterns, respectively. These representations were preserved at the outputs of the NPLLs (Figure 7B, SC horizontal).

**Figure 8:**
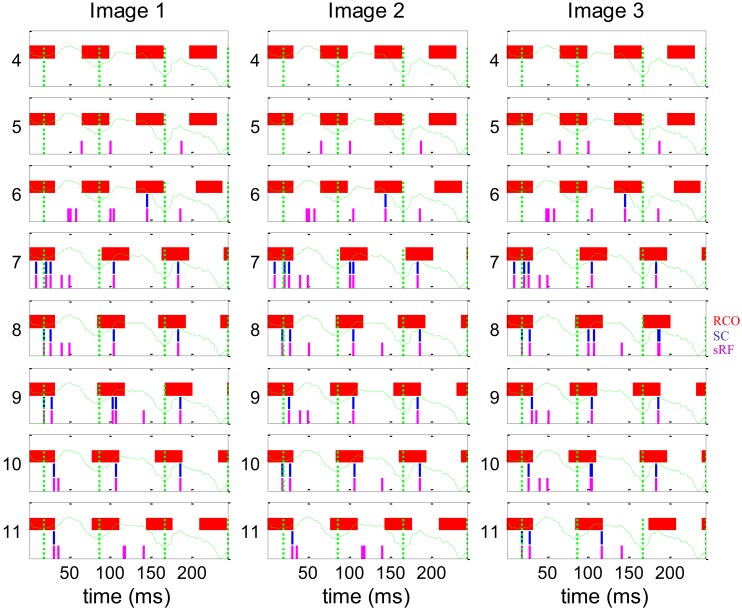
**Dynamics of the encoding-decoding process**. The spike trains of RCO (red vertical lines), SC (blue vertical lines), and sRF (magenta vertical lines) units are shown for the simulations presented in Figure 7. The horizontal trace of FeyeM is depicted in green. Vertical dotted green lines mark cycle onset. Eight units (*y* = 4–11) are depicted along one column (*x* = 10). Conduction delays were simulated as zero. Initial RCO frequency was 0.8 of the mean horizontal FeyeM frequency (12.3 Hz) and the open-loop gain was −5.

The dynamics of spike generation in relevant horizontal NPLLs are depicted in Figure 8. The four coding NPLLs (#7–10) received sRF inputs that allowed phase-locking to the horizontal FeyeM. As a result, the RCO bursts (red vertical lines) were phase-locked to the FeyeM cycle onsets (dotted green vertical lines). This resulted in a reduced frequency in the RCOs of these NPPLs: from 4 to ∼3 cycles in the time window depicted. RCO locking to FeyeM enabled the translation of sRF firings (magenta vertical lines) to SC firings (blue vertical lines). The other four NPLLs fired out of phase with the input and thus blocked their corresponding sRF signals.

These computer simulations demonstrate the validity of temporal encoding of shape by FeyeM, the superiority of temporal coding along over rate coding across the elongated axes of sRFs for fine spatial details, and the ability of NPLLs to phase-lock to retinal outputs, and thus recode shape by relative time coding, with natural FeyeM. These simulations do not address encoding-decoding of object motion or texture.

### How we see a stable world via trembling eyes

One long-standing unresolved puzzle in vision is: how come the world appears stable if our eyes move all the time? The answer to this puzzle is surprisingly simple. This puzzle exists *only* if one assumes that FeyeM are not part of the visual mechanism. For example, if the visual system would read a spatial retinal code, this code would move across the retina according to the trajectory of FeyeM. In that case, some mechanisms in the brain should correct for eye movements, and it is totally unclear what mechanism could do that. However, if vision is done via FeyeM, no correction is needed. These very movements *sample* the information in; this is the way the visual system acquires information, exactly as the tactual system acquires information by moving the fingers (or the whiskers in rodents). As the system is tuned for moving its sensors to acquire information, there should be nothing in the system that interprets a coherent movement of the entire sensory field as a movement of the world. Information about the actual movement of the eye is continuously conveyed to the brain by the spatiotemporal activation of horizontal and vertical retinal sRFs (see Encoding of FeyeM Direction and Resolving Image Details by sRF Structure: 1D Vision above). Interference with this spatiotemporal pattern contributes to motion illusions such as autokinesis and induced movement (Poletti et al., 2010), suggesting their inclusion in the seeing loop. A prediction of seeing via FeyeM is thus that small or slow FeyeM-like movements of the entire visual field should not be perceived [see prediction (ii) below].

### The visual motor-sensory loop

**Figure 9:**
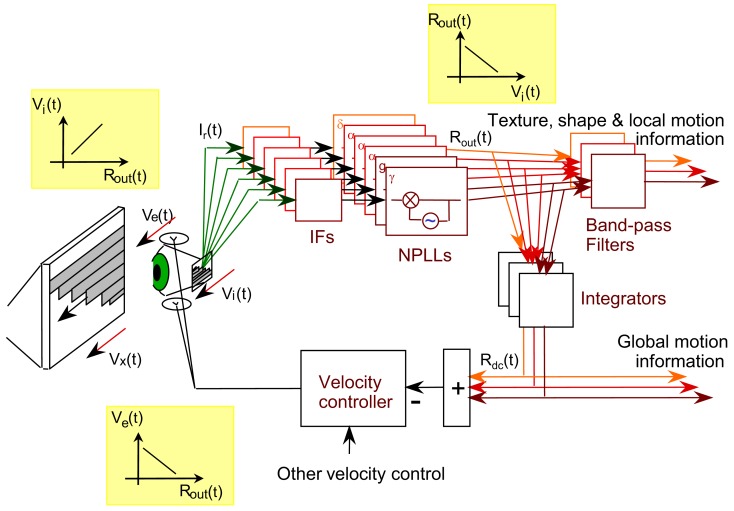
**Schematic diagram of a motor-sensory visual loop containing a bank of NPLLs of different intrinsic frequencies**. *V_x_*, *V_e_*, *V_i_*, velocities of an external object, the eye, and the retinal image, respectively; IF, intermediate filter that is centered around the working frequency of its corresponding NPLL; δ, α, γ, the frequency range containing the intrinsic frequency of an NPLL. The major transfer functions dominating the loop are described in the three insets: *R*_out_ is the output firing rate of an NPLL.

Optimal functioning of NPLLs requires their operation within a motor-sensory feedback loop (Ahissar and Vaadia, 1990; Ahissar, 1998). Schematically, the loop level containing the NPLLs can be described as is shown in Figure 9. The movements of the eye [*V_e_*(*t*)] and external objects [*V_x_*(*t*)] induce movements of the image on the retina [*V_i_*(*t*)] which generate afferent visual signals (green arrows). The afferent signals are filtered along their afferent pathway (IFs) and fed into thalamocortical NPLLs. The loop contains many NPLLs, each tuned to a different intrinsic frequency and thus capable of decoding visual information carried by a different range of temporal frequencies. Available data on thalamocortical visual oscillations suggest that this bank of NPLLs span the range of frequencies between 1 and 100 Hz, with some tendency for frequencies in the δ, α, and γ ranges (Gray et al., 1989; Eckhorn, 1994; Gray and McCormick, 1996; Brumberg et al., 2000; Cardin et al., 2005; Bosman et al., 2009). The outputs of the NPLLs drive circuits that decode shape, texture and local motion information, and in addition close the motor-sensory loop by driving motor circuits controlling eye velocity. This entire motor-sensory loop functions as a negative feedback loop; a change in eye velocity induces a change in NPLLs outputs [*R*_out_(*t*)] that generates motor signals that oppose the change in eye velocity (insets of transfer functions). By monitoring the mean output of NPLLs and modifying eye velocity accordingly such a negative feedback loop can maintain the mean temporal frequency of the retinal output in a desired range (The Motor-Sensory Loop in Appendix 1).

At any given viewing period there should be one or several NPLLs, out of the many NPLLs included in the bank, that dominate the motor-sensory loop; the motor-sensory loop functions to optimize the input for these NPLLs during that period. Given the dynamics of frequency variation in FeyeM (e.g., Moller et al., 2002) it would make sense to assume a dynamic shift of control from one pool of NPLLs to another every three or four FeyeM cycles. With optimal tuning, such a period should be sufficient for efficient temporal decoding (Figures 7 and 8; Ahissar, 1998). The mechanism that determines the shift of control within the NPLLs bank is not yet clear, although a few potential principles for its operation had been suggested (Ahissar, 1998).

## Discussion

Natural vision is a continuous process that, at any given moment, has to deal with retinal activity that has been affected by recent FeyeM and by optical blurring. FeyeM enforce temporal encoding at the retina. Spikes of retinal outputs indicate times in which image contrasts are crossed by moving receptors, much like spikes of mechanoreceptors indicate times in which their RF crosses a ridge. Such temporal encoding has a hyperacuity resolution and is resistant to optical blurring. Metaphorically, vision can be described as an active process in which the retina “palpates” external objects like rat whiskers and human fingers do. In both vision and touch, the sensory organs are mainly activated when they encounter changes during their scanning movements.

We suggest here a specific way in which the visual system could decode temporally encoded retinal signals. The proposed active decoding scheme involves cortical oscillations (which function as “temporal rulers”) and thalamic phase comparators (which compare input timing against the “ruler”). This scheme is consistent with a large body of anatomical and physiological data. Although the data supporting it are compelling, we do not claim that this proposed scheme is the only possible decoding mechanism or that there is a single decoding mechanism for retinal outputs. Several such mechanisms are available to the visual system, and brains must emphasize one or another mechanism, depending on the visual stimulus, context, and previous experience. During natural viewing, we suggest, the active decoding mechanism described here plays a major role.

### Advantages and limitations of the proposed encoding-decoding scheme

The temporal encoding-decoding scheme presented here provides a mechanism that is free from retinal smearing that would otherwise be caused by FeyeM. According to this scheme, local hyperacuity is resistant to optical blurring because of the differential nature of retinal temporal encoding. Utilization of the temporal domain by the visual system allows information to be accumulated, rather than averaged, during the entire fixation period. Furthermore, such utilization of temporal coding enables control of visual resolution by simply controlling eye velocity (Saig et al., 2012); such control can be used to adapt visual resolution to the spatial frequencies of the external image. The decoding by intrinsic oscillations provides an adaptive band-pass filtering mechanism in which the center frequency of the filter tracks the dominant frequency in the input. Note that this mechanism provides also a “temporal extrapolation” mechanism, which can predict the time in which a moving object will cross a given retinal RF by locking to the Doppler-shifted frequency (see *Phase Locking* above). Finally, decoding by intrinsic oscillations provides internal temporal markers that can facilitate serial processing of sequential chunks of information, chunks that contain inter-related information such as the information sampled in parallel across the retina during each FeyeM cycle.

Accurate vision, if mediated by NPLLs, should require continuous fixation. This is not inconsistent with common experience and controlled studies indicating that visual acuity does improve with longer fixational period (Riggs et al., 1953; Keesey, 1960; Morgan et al., 1983; Packer and Williams, 1992). Following a stimulus onset, visual accuracy is incrementally impaired, and starts to improve again only after about 200 ms (Packer and Williams, 1992). Interestingly, image stabilization during the initial periods of fixation facilitates accurate vision. Beyond ∼100 ms, stabilization is detrimental and retinal motion is helpful for accurate vision (Riggs et al., 1953). All these data are consistent with visual accuracy being impaired by the smearing caused by FeyeM until thalamocortical circuits lock-in and start to decode the temporally encoded information. According to this scenario, it makes sense for the visual system to ignore cortical outputs immediately after a saccade for periods up to about 200 ms, a phenomenon known as saccadic suppression (Carpenter, 1988).

### Complications and challenges

To prevent aliasing (which occurs when the sampling frequency is too close to the sampled frequency) temporal encoding should, and probably does, rely on multiple frequencies (see Ahissar and Arieli, 2001). During a fixation period, different frequencies can be superimposed or can appear sequentially. Based on accumulated data (Examples of Human FeyeM in Appendix 2 and published FeyeM traces), our decoding example (Figure 5) assumed the latter. However, when different FeyeM frequencies are superimposed, and head movements are added (Steinman and Levinson, 1990), the eye spatially drifts, and consequent cycles do not start at the same retinal location. Thus, thalamocortical circuits should be coupled such that locking information propagates through neighboring circuits (Hoppensteadt and Izhikevich, 1998). In fact, if coupling strengths can change, networks of NPLLs can learn to process complex patterns of oscillatory inputs (Hoppensteadt and Izhikevich, 2000), generated by various interactions of FeyeM with external images. If efferent copies of FeyeM exist, they can significantly facilitate this process (as well as facilitating initial locking-in after saccades). In any case, the challenge of a continuous processing of drifting retinal image is not unique to the present model – it is a general challenge, which every model of vision must deal with.

In fact, the complication induced by slow eye drifts can be better handled by temporal decoders of the type presented here than by spatial decoders that are based on cell identity. Since temporal changes induced by eye drifts are common to the entire visual field, they can be decoded by widely tuned NPLLs, low-pass integration of NPLL outputs (Ahissar, 1998), or by other specialized networks (e.g., Pitkow et al., 2007). Once decoded, this information can be used to control the coupling between neighboring fine-tuned NPLLs (Ahissar, 1998). Note that in any case this global drift, being common to all NPLLs, should not distort the differential temporal coding of local spatial details described here.

Reading out cortical differential representations should involve lateral comparisons of outputs of simple cells, which could be ambiguous if the response polarities (i.e., ON vs. OFF) of the compared cells are not known. This problem is probably circumvented by segregation of thalamocortical circuits to ON-center and OFF-center clusters (McConnell and LeVay, 1984; Lund et al., 1985; Zahs and Stryker, 1988); within each such cluster response timing can be compared across cells with a remarkably high precision (Reinagel and Reid, 2002). A similar segregation, according to color sensitivity, is expected to circumvent ambiguities due to chromatic aberration. Such segregation was observed in the human retina (Roorda and Williams, 1999).

Cortical (and retinal) representations of external velocities are unique only if the amplitude of the FeyeM is smaller than both the sRF length and the external spatial periods. Otherwise, the transformation is not unique; different combinations of external spatial periods and external velocities could induce similar cortical spike counts. When the sRF is longer than the external spatial periods, aliasing problems are introduced, which cause additional ambiguities. The visual system could avoid such ambiguous coding by relying on high frequency low-amplitude FeyeM for foveal vision, and on FeyeM with increasing amplitudes (associated with decreasing frequencies) for increasingly eccentric vision. The finding that rod monochromat subjects, who lack foveal receptors, exhibit large-amplitude-low-frequency nystagmus (Yarbus, 1967; pp. 119–122) is in agreement with such a scheme, which suggests that the lack of foveal reception eliminates the need for high frequency FeyeM, leaving only low frequency FeyeM, which become more coherent. Further support comes from stabilization experiments that showed that when only large-amplitude movements are compensated, only peripheral vision fades away (Gerrits, 1978).

During natural fixation, drift, and tremor movements are often interrupted by brief microsaccades (see Introduction), which bounce retinal RFs to new locations. Obviously, computations of 2D details by NPLLs cannot continue across microsaccades, and should not include data acquired during microsaccades. Interestingly, many cortical neurons respond either during microsaccades (“saccade cells”) or during drift (“position/drift cells”) but not during both (Snodderly et al., 2001). This indicates that the visual system contains independent channels that enable independent processing of visual information acquired during drift-tremor and during microsaccades.

### A global view on the perceptual process

Every sensory system contains multiple motor-sensory loops that together perceive components of the external world. Such loops contain sub-cortical and cortical stations and pathways which interact in complex ways (see for example the scheme of the vibrissal motor-sensory system in Kleinfeld et al., 2006). The encoding-decoding scheme proposed here belongs to an intermediate level of the visual motor-sensory system; there are lower loops that control more basic functions and higher loops that interpret the outputs of the circuits described here. At all loop levels, the processed sensory information affects next eye movements which in turn affect future sensory inputs (Figure 9; see also Uchida et al., 2006). The current paper focuses on the encoding and decoding of temporally coded information generated by FeyeM. However, it is assumed that this processing is one component of the entire system of motor-sensory loops composing the visual system, and that this entire system works in coordination when perceiving external objects. Thus, the interpretations of the outputs of the circuits described here are components in the working of higher (and perhaps also lower) motor-sensory visual loops. Another assumption is that at each level there are many parallel circuits and loops that either compete or cooperate in controlling motor functions, depending on the conditions. According to this scheme, the chaotic-like patterns of eye movements typically observed reflect superposition of many competing and cooperating processes implemented along many motor-sensory loops. How, eventually, a coherent perception emerges from such a seemingly messy process is of course not yet clear. Still, the leading assumption here is that although the process looks chaotic to an external observer, there is a hidden order here as each of the operating loops is tuned to process the outcome of its own controlled components of eye movements.

### Are periodic FeyeM critical for the proposed scheme?

We describe the principles of the encoding-decoding scheme using examples of periodic FeyeM (e.g., Figures 3 and 5). Still, pure periodicity is not a critical requirement of the proposed scheme. First, as explained in Section “*Phase Locking*” above, and shown in the Section “Loop Dynamics” in Appendix 1, the decoding circuit can track changes in the input frequency up to a certain limit (see also Ahissar, 1998; Zacksenhouse and Ahissar, 2006). Second, the elongated structure of sRF allows also large tolerance for amplitude modulations: when the amplitude of the FeyeM changes, the temporal information is not affected; the only changes are in the number of sRFs that represent the stimulus and in the number of spikes conveyed by the FF signals. Since these changes are global to the entire retina, relative coding will hardly be affected. Similarly, the component of FeyeM that is perpendicular to the elongated axis of the sRF does not impair temporal coding. In fact, this perpendicular drift allows scanning of many different alignments between sRFs and a given spatial offset in the image. Thirdly, we propose that every decoding loop processes sensory information in a relatively narrow band (about one octave around its intrinsic frequency), and ignores fluctuations outside this band (see The Visual Motor-Sensory Loop above). The simulations with natural FeyeM presented in Figures 7 and 8, and simulations with amplitude and frequency modulated synthetic FeyeM (not shown) demonstrate that temporal coding functions fairly well with repetitive FeyeM that deviate significantly from pure periodicity.

### Consistency with other relevant experimental data

Electrophysiological and anatomical:

- In some conditions, cortical alpha oscillations correlate with ocular oscillations (Lippold, 1973). In other conditions, no or small correlation is observed (Butler and Glass, 1970). Our hypothesis assumes that the ocular and cortical oscillatory sources are independent sources that become coupled during vision. Accordingly, the cortical alpha rhythm usually indicates an idling state (in which cortical oscillators phase-lock to each other), a state that disappears when the eyes scan external patterns [and each oscillator locks to the temporal pattern of its specific input (Ahissar and Vaadia, 1990); recent fMRI data support cortical idling during alpha spindles (Feige et al., 2005)]. During such scanning, correlations between FeyeM and local cortical activity are expected, although they should not necessarily be periodic, and should not necessarily be correlated across the cortex.
- Visual cortical activity can be locked to a periodic visual stimulus (a phenomenon called “photic driving,” Walter and Walter, 1949) in a fashion supporting a resonance-like process between the visual stimulus and multiple local oscillators (Tyler et al., 1978; Fedotchev et al., 1990; Basar et al., 1991; Schurmann and Basar, 1994). The model suggested here behaves exactly in this manner – a population of local oscillators resonates with the temporal rhythm of the retinal signal while decoding phase variations.
- Visual cortical activity can remain “locked” to the stimulus frequency after the cessation of the stimulus (Narici et al., 1987; Sakamoto et al., 1993). This “temporal memory” requires a closed loop operation, either at the cellular level, or at the circuit level as suggested by the NPLL model.
- Retinal, thalamic, and cortical neurons exhibit phase-locked activity during stimulus presentations, during which cortical neurons often phase-lead thalamic neurons (Castelo-Branco et al., 1998), as predicted by the NPLL model.
- Frequencies of cortical oscillations change when stimulus velocity changes (Eckhorn et al., 1993; Gray and Viana Di Prisco, 1997), as predicted by the Doppler-shift of the sampling frequency.
- Retinal spike times convey more visual information than spike counts (Berry et al., 1997).
- Thalamic transfer and timing depends on cortical feedback (reviewed in Rauschecker, 1998).
- Responses of cat retinal ganglion cells to tiny (= cone separation) retinal motion are locked to movement onset (Shapley and Victor, 1986).

Psychophysical:

- Temporal aliasing occurs during daylight vision (Purves et al., 1996; Pakarian and Yasamy, 2003; Andrews and Purves, 2005; VanRullen, 2006), which indicates a sampling process in time.
- Metacontrast: consequent non-overlapping visual stimuli cannot be perceived in isolation for temporal intervals smaller than 50–150 ms (Bachmann, 1997; Ogmen et al., 2003). This is consistent with an alpha rhythm based sampling process.
- Flicker fusion is not integrated between the eyes (Andrews et al., 1996), which indicates that temporal processing is monocular, as required by our proposed scheme, due to lack of synchronization of FeyeM between the two eyes.
- Movement processing is often based on dot elements rather than on line elements, i.e., on two-dimensional discontinuities such as corners, intersections, and endpoints of contours (Rubin et al., 1995; Caudek and Rubin, 2001; Pack et al., 2003b). This is consistent in principle with processing along the elongated axis of sRFs. The perception of a moving dot exhibits sensitivity and attributes that are similar to those exhibited by perception of solid lines (Westheimer and Wehrhahn, 1994).
- Motion smear disappears once motion is perceived (Burr, 1980), possibly due to locking-in of thalamocortical loops. During apparent motion, temporal delays are perceived as spatial offsets (Burr, 1979; Fahle and Poggio, 1981), as suggested here.
- In some experiments, 2D acuity was found to be limited by temporal differential delays, not by spatial offsets or velocities (Carney et al., 1995). In other experiments, spatial offset appeared to be the limiting variable. Threshold temporal delays are in the order of 1 ms (Burr, 1979; Fahle and Poggio, 1981; Morgan and Watt, 1983; Carney et al., 1995), which correspond to the temporal accuracy of visual signals up to the thalamic level (Levick et al., 1972; Lee et al., 1981; Berry et al., 1997). Moreover, both acuity thresholds and temporal uncertainties had been shown to follow power-law functions with similar exponents (Wilson, 1986; Berry et al., 1997; Verdon-Roe et al., 2006). These findings are consistent with the scheme suggested here of a serial temporal-spatial processing implemented by thalamocortical and corticocortical circuits, respectively.
- Hyperacuity thresholds are not affected by retinal image degradation (Williams et al., 1984), indicating that relative resolution does not depend on accumulation of firing rates over space.
- Temporal asynchrony interferes with Vernier acuity. Judgment of vertical alignment of two dots is impaired if the two dots are not presented synchronously (Wehrhahn and Westheimer, 1993).
- Elimination of retinal motion induced by FeyeM selectively impairs the discrimination of fine spatial details, while leaving the discrimination of coarse spatial details unaffected (Rucci et al., 2007).
- Humans show power-law dependency on the stimulus contrast in various accuracy and hyper-accuracy tasks, with exponents similar to those observed at the retina (−0.5 to −1; Wilson, 1986; Verdon-Roe et al., 2006); the power-law dependency holds up to 100% contrast. These dependencies are similar to the dependency of retinal jitter on stimulus contrast (Berry et al., 1997) which supports a dependency of visual acuity on retinal temporal jitter.
- EEG recordings reveal that (i) neuronal oscillations code sensory information relevant for visual perception, (ii) frequency, phase, and amplitude play differential roles in coding behaviorally relevant information in the brain, and (iii) phase contain higher information than power (Schyns et al., 2011).

Alleged inconsistencies with relevant experimental data:

- Keesey (1960) and others found that stabilization does not impair spatial acuity. However, it turned out that images probably cannot be fully stabilized (Barlow, 1963; Steinman and Levinson, 1990) and that acuity is gradually impaired as stabilization improves (Kelly, 1979). With good stabilization, and conditions of natural viewing, visual discrimination is consistently and significantly impaired (Rucci and Desbordes, 2003).
- Vernier acuity of 2 ms flashed stimuli is as good as that of longer stimuli provided that the intensity × duration product is constant (Hadani et al., 1984). This could imply that (i) temporal information, and FeyeM in general, is not required for detecting fine offsets when flashed on the retina, or (ii) temporal information can be extracted from afterimages that are moved across non-homogeneous backgrounds. However, since the thresholds obtained in the “constant energy” condition were higher than the minimal thresholds obtained (during long durations) by the two subjects that were tested, and since one of them had a threshold >10,” we find these results not conclusive. In any case, detection of offsets in flashed stimuli utilizes visual mechanisms in a non-natural way. Naturally, visual mechanisms must deal with continuous, non-flashed, stimuli which are traversed by the eyes. That hyper-accurate detection is also possible with some artificial conditions is of course possible.

### Predictions for active vision

Critical for temporal encoding at the retina:

(i) Small or slow movements (with amplitudes or velocities smaller than those of FeyeM) of the entire image should not impair local (hyper) acuity. However, spatially-non-coherent movements of details of the image, even if their average locations are kept constant, should.
(ii) Small or slow movements (with amplitudes or velocities smaller than those of FeyeM) of the entire visual field should not be perceived, while spatially-non-coherent movements should.
(iii) Synchronous temporal fluctuations of image intensity should not impair local acuity, while asynchronous fluctuations of details of the image, even if their locations are kept constant, should.
(iv) Retinal latencies depend on contrast (Gawne et al., 1996). Thus, contrast differences should be perceived as spatial offsets also with stationary stimuli, and not only with moving stimuli (e.g., Williams and Lit, 1983). This of course depends on the direction of the FeyeM during the viewing epoch. For example, if one line of a vernier is presented with a higher contrast, it should be perceived as relatively positioned more leftward when the eye moves to the right.

Critical for decoding by thalamocortical NPLLs:

(v) Cortical simple cells should represent local spatial-phase relationships (i.e., fine determinants of shape and texture) by temporal phase relationships, and relative velocities by relative spike counts.
(vi) When the temporal frequency of the retinal output increases, retino-cortical delays should increase, and the spike counts of simple cells should decrease.

Non-critical:

(vii) If FeyeM can be centrally controlled (Shakhnovich, 1977; Coakley, 1983; Eizenman et al., 1985), subjects should modify the frequencies, amplitudes, and/or velocities of their FeyeM according to the spatial frequency content of the image.
(viii) Such adaptations should occur during long fixations (>200 ms), and are expected to stabilize at conditions that produce temporal frequencies (at the retinal output) within the alpha or gamma ranges, which are probably preferred by thalamocortical loops.
(ix) Opening eyes in full darkness, or against a uniform image, should not desynchronize cortical EEG. Cortical EEG is expected to desynchronize only when viewing a patterned image, in which case different cortical oscillators are expected to track different temporal patterns.
(x) End stopping: end-stopped cells are tuned for bar lengths: they are excited by short bars and are being gradually inhibited as the length of the bar increases (see, e.g., Bolz and Gilbert, 1986). This inhibition is thought to originate in oriented cortical cells. If these inhibitory oriented cells are simple cells that are involved in NPLLs (Figure 6), their output would depend on the frequency. Thus, when retino-cortical phase-locking is maintained, e.g., during natural viewing, as the input temporal frequency increases toward the cortical intrinsic idle frequency, end-stopped cells are expected to gradually become less inhibited, and the latency of their inhibition to gradually increase.
(xi) Snodderly’s “position/drift cells” (Snodderly et al., 2001) should be sensitive to 2D spatial relationships and motion velocity whereas “saccade cells” should be sensitive to binocular disparities.

Additional predictions are described in Ahissar (1998).

## Conflict of Interest Statement

The authors declare that the research was conducted in the absence of any commercial or financial relationships that could be construed as a potential conflict of interest.

## Figures and Tables

**Figure A1:**
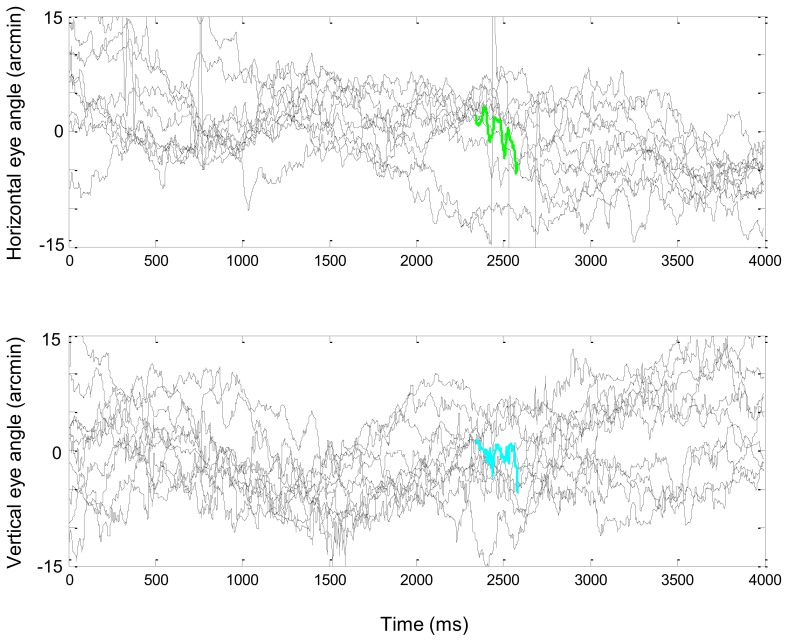
**FeyeM recorded from one human subject while fixating on a single point distanced 1 m from the subject**. Ten repetitions of a 4-s fixation period are shown. The three-cycle epoch used in the simulations of **Figures 7** and **8** are colored in green and cyan. Courtesy of Dr. Moshe Fried.

**Figure A3:**
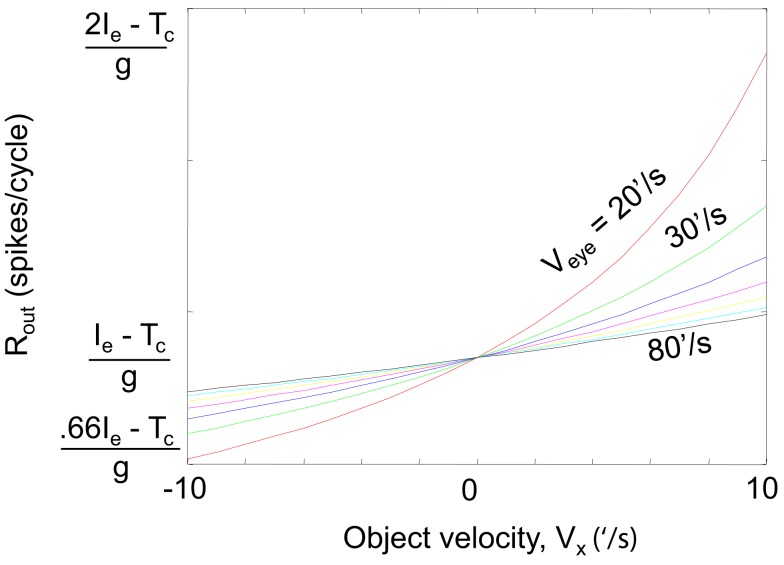
**Dependency of *R*_out_ on image velocity (*V*_x_)**. Equation A10 in Appendix 1 is plotted for *V*_eye_ = 20–80’/s, *I*_eye_ (*I*_e_) = 100 ms, and *T*_c_ = 66 ms.

## Appendix 1

### Temporal encoding

A given spatial offset in a stationary image, Δ_x_, is translated to a neuronal temporal delay, Δ*t*, by the velocity of the eye in the same direction, *V*_eye_, such that:

(A1) $$\Delta_{t}=\Delta_{x}∕V_{\text{eye}}\text{,}$$

for clarity, we ignore here the dependence of these variables on time.

### Retinal frequencies and encoding of motion

Determination of retinal frequencies depends on the relationship between the texture of the image (*S*_x_, spatial period) and the peak-to-peak amplitude of FeyeM (*A*_eye_). We will limit our discussion to cases in which the amplitude of eye movement is smaller than a single cycle of contrast change (*S*_x_ > *A*_eye_). In these cases, the retinal periodicity (*I*_r_) follows the periodicity of the FeyeM (*I*_eye_):

(A2) $$I_{\text{r}}=I_{\text{eye}}$$

Note that due to the dependency of *A*_eye_ on *I*_eye_ (see Ahissar and Arieli, 2001), different spatial periods will fulfill the above criterion (*S*_x_ > *A*_eye_) at different FeyeM frequencies (see also Discussion: Complications and Challenges).

With moving images, the resulting retinal period for a given sRF will be:

(A3) $$I_{\text{r}}=I_{\text{eye}}\cdot \frac{V_{\text{eye}}}{V_{\text{eye}}-V_{\text{x}}},\;\text{for}\left|V_{\text{x}}\right|<\left|V_{\text{eye}}\right|$$

where *V*_eye_ represents the velocity of the eye in the protraction direction (approximated to be constant here), and *V*_x_ is the external velocity in the same direction (Figure 3D). We will not deal here with |*V*_x_| ≥ |*V*_eye_|.

### Cortical oscillations

We use a linear version of the equation presented by Perkel et al. (1964) to describe the control of neuronal oscillations via inhibitory cells.

(A4) $$I_{\text{o}}\left(n\right)=T_{\text{c}}+\gamma R_{\text{out}}\left(n-1\right)$$

where *I*_o_(*n*) is the oscillation period at cycle n, *R*_out_(*n−*1) is the number of spikes driving the inhibitory neurons during cycle *n−*1, and *T*_c_ is the intrinsic oscillation period (i.e., the oscillation period when *R*_out_ = 0). The constant γ, whose units are ms/spikes, determines the gain of this control.

### Thalamic phase detection

In the gating mode, thalamic neurons fire only when the retinal and cortical inputs overlap. Thalamic output would be maximal when the two inputs fully overlap (τ*_D_* = 0, where τ*_D_* is the delay between the two inputs), and will gradually decrease as the delay increases until no overlap occurs. If the gradual decrease is linear, the thalamic PD can be approximated as:

(A5) $$R_{\text{out}}\left(n\right)=\left\{\begin{array}{cc} R-\alpha \tau_{\text{D}}\left(n\right),\; & \text{for}\;R>\alpha \tau_{\text{D}}\left(n\right)\\ 0,\; & \text{otherwise} \end{array}\right.$$

where *R*_out_(*n*) is the number of spikes generated by the PD during cycle *n*, *R* is the maximal number of spikes that can be generated by the PD [when τ_D_(*n*) = 0], and α is the gain of this function. *R*, and possibly also α, depend on the contrast; this dependency is ignored in the present discussion. The output signal is expressed in units of spike count, and thus cannot be assigned non-integer values; for clarity, this limitation is omitted from the equation.

### Loop operation

The retino-cortical delay at cycle *n*, τ_D_(*n*), depends on the delay at cycle *n−*1 and on the difference between the retinal [*I*_r_(*n*)] and cortical [*I*_o_(*n*)] periods at cycle n:

(A6) $$\tau_{\text{D}}\left(n\right)=\tau_{\text{D}}\left(n-1\right)+I_{o}\left(n\right)-I_{r}\left(n\right)$$

This equation shows that the only condition in which τ_D_(*n*) = τ_D_(*n−*1), namely that the loop is at a steady-state, is when the oscillation period equals the retinal period:

(A7) $$I_{\text{o}}\left(n\right)=I_{\text{r}}\left(n\right)$$

The combination of Eqs 4, 5, and 7 reveals the dependency of the set point, i.e., the values of τ_D_ and *R*_out_, on the input frequency:

(A8) $$\tau_{D}=\frac{R}{\alpha }-\frac{T_{\text{c}}-I_{\text{r}}}{G},\;G=-\alpha \;\gamma$$

where *G* is the “open-loop gain,” which equals the multiplication of the local gains along the loop. *R*_out_ at steady-state can be determined by combining Eqs 5 and 8:

(A9) $$R_{\text{out}}=\frac{I_{\text{r}}-T_{\text{c}}}{\gamma }$$

Thus, *R*_out_ directly represents *I*_r_, and thus also *V*_x_ (from Eqs 3 and 9, at steady-state):

$$\begin{array}{llll} \gamma R_{\text{out}}=I_{r}-T_{c} & =\left[\frac{\left(I_{\text{eye}}-T_{c}\right)V_{\text{eye}}+T_{c}V_{x}}{V_{\text{eye}}-V_{x}}\right] & & \\ & =\frac{I_{\text{eye}}-T_{c}+I_{\text{eye}}V_{x}}{V_{\text{eye}}-V_{x}},\;\text{for}|V_{x}|<|V_{\text{eye}}| & & \text{(A10)} \end{array}$$

If *V*_x_ = 0, *R*_out_ represents the frequency of eye movements, or more accurately, the deviation of the eye frequency (1/*I*_eye_) from the NPLL’s intrinsic frequency (1/*T*_c_). If |*V*_x_| < |*V*_eye_|, and the image component is moving in the protraction direction, *V*_x_ is positive and *R*_out_ will increase (compared to a stationary image). If it is moving in the retraction direction, as in Figure 5A, *R*_out_ will decrease, as in Figure 5E. The dependency of *R*_out_ on *V*_x_, for various *V*_eye_ values, is described in Dependency of *R*_out_ on Image Velocity in Appendix 2.

The loop can converge on a limited set of input frequencies; a set termed “the working range”. The working range depends on *G*: it is empty for *G* ≥ 0, and increases as *G* becomes more and more negative. Thus, for all loops in which the working range is not empty *G* < 0 and τ_D_ decreases with retinal periodicity (*I*_r_; see Eq. 8), or increases with retinal frequency (1/*I*_r_; Figure 6B).

### Loop dynamics

The dynamics of the loop while tracking can be revealed by combining Eqs 4–6:

(A11) $$\tau_{D}\left(n\right)=T_{c}+\gamma R+\left(1+G\right)\tau_{\text{D}}\left(n-1\right)-I_{r}\left(n\right)$$

The only case in which τ*_D_*(*n*) does not depend on τ*_D_*(*n−*1), i.e., on the history of the loop, is when *G* = −1. In this latter case, the loop can track changes in the input periodicity with a delay of only a single cycle (see the case depicted in Figure 5D). As *G* diverges from −1, tracking becomes less and less accurate, since convergence to a fix point takes more and more cycles (Ahissar, 1998).

### Cortical representations

Retinal periodicities [*I*_r_(*n*)] are translated into latencies [τ*_D_*(*n*); Eqs 8 and 11] and spike counts [*R*_out_(*n*); Eq. 10] of cortical simple cells (Figure 6B). The steady-state cortical representations are described by Eqs 8 and 9. These cortical representations should be valid at the input layers of *V*1, and of course might be further transformed in successive processing levels.

These cortical (and retinal) representations depend on the ratios between the amplitude of the FeyeM (*A*_eye_), the sRF length (*L*_RF_), and the external spatial periods (*S*_x_ = 1/spatial frequency). In this paper we focused on cases in which *A*_eye_ < *S*_x_ and *L*_RF_ < *S*_x_. In these cases, external velocities (*V*_x_) are represented by cortical spike counts. In cases in which *A*_eye_ > *S*_x_, the transformation is not unique; different combinations of *S*_x_ and *V*_x_ could induce similar spike counts. In cases in which *L*_RF_ > *S*_x_, aliasing problems are introduced, which cause additional ambiguities. There is probably enough information available to the visual system to resolve all these ambiguities by utilizing intra-burst frequencies, lateral comparisons, and orientations of sRFs. Alternatively, the visual system could avoid this ambiguous coding by relying only on those thalamocortical circuits for which, in a given epoch, *A*_eye_ ≈ *L*_RF_ < *S*_x_ (see Discussion: Complications and challenges).

### The motor-sensory loop

The visual motor-sensory loop (Figure 9) controls the input periodicity by monitoring the output of NPLLs and modifying eye velocity accordingly. The loop equations are:

$$\begin{array}{rcll} & V_{e}\left(t\right)=g_{\text{v}}\left(R_{\text{max}}-R_{\text{dc}}\left(t\right)\right)+V_{\text{other}} & & \text{(12)}\\ & R_{\text{dc}}\left(t\right)=g_{\text{pll}}\left(I_{r}\left(t\right)-T_{c}\right) & & \text{(13)} \end{array}$$

where *V*_e_(*t*) is the velocity of the eye in one dimension (e.g., horizontal), *g*_pll_ and *g*_v_ are the NPLL’s and velocity controller’s transfer functions, respectively (Figure 9), *I_r_*(*t*) is the inter-spike interval of the retinal output, *T*_c_ is the NPLL’s intrinsic period, *R*_dc_(*t*) is the mean integrated output of NPLLs, *R*_max_ is the maximal possible *R*_dc_(*t*), and *V*_other_ is the velocity additive component caused by the “Other velocity control” input.

## Appendix 2

### A simple quantitative account against spatial retinal coding of fine spatial details

Our ability to detect relative offsets is an order of magnitude better than the separation power of the eye. For example, we can detect an offset of a few arcseconds between two co-linear lines (vernier hyperacuity) while we can detect a separation between two parallel lines only if they are separated by about an arcminute (separation acuity; Westheimer, 1976). Hyperacuity is achieved only in relative coordinates, i.e., only between co-existing elements (McKee and Levi, 1987; McKee et al., 1990). It had been shown that separation acuity is limited by the amount of smearing caused by the optics of the eye and by cone spacing in the fovea – two variables that match each other (Westheimer, 1987). It is not known what limits hyperacuity and how it is achieved. It is widely assumed that hyperacuity benefits from the very same factor that limits separation acuity – optical smearing. Since the optics of the eye spread each tiny spot of light over several cones, the location of that spot can be computed by computing the center of cone activation. The resolution of such localization does not depend on cone granularity but on the granularity of cone responses, i.e., by the power of cone response in resolving the intensity of light absorbed by it.

Thus, with the aid of optical smearing, and perhaps overlapping RFs, the challenge of obtaining hyperacuity resolution can be transferred to the intensity domain. However, with all-or-none spikes, response intensity can only be measured along time. How much time is required for collecting enough spikes to detect a vernier offset? A retinal network that is simulated under realistic conditions can generate a one to two spikes difference for a near-threshold vernier offset, if it is given 60 ms (Wachtler et al., 1996). To check whether this time period depends on the actual detection mechanism, we performed a simple calculation. We examined the time required for generating a one spike difference in the ensemble firing probability, where the only assumption is that the response of each cell in the population decreases monotonically with the visual angle of the stimulus. Interestingly, this time window hardly depends on the size of the population, and depends only on the maximal firing rate evoked in that population and on the vernier offset (Spatial Encoding of Hyperacuity by Firing Rates in Appendix 3). With realistic response values (Wachtler et al., 1996) and an offset of 1/4 of a ganglion RF diameter, this time window is estimated as ≥80 ms. This time window could decrease significantly had the retinal signal been duplicated over several such ganglion populations. However, vernier acuity hardly improves when the vernier components are elongated. In fact, excellent vernier acuity is obtained with just two dots. This observation by itself already contradicts simple accumulation of retinal firing rates, or of other pure spatial signals, as a mechanism for hyperacuity.

The main consideration that invalidates a pure spatial encoding for fine details is the following. As the above observations show, it would take 60 ms or more to obtain a minimally detectable difference in spike counts of ganglion cells, whose RF diameters are smaller than 1 arcmin (foveal RFs get as small as 0.3 arcmin). However, during these 60 ms the eye had traveled over a significant number of such RFs [estimations would vary between 2 and 10 arcmin, equivalent to 6–30 RFs, depending on the study: (Ratliff and Riggs, 1950; Barlow, 1952; Moller et al., 2002; Cherici et al., 2012; see Figures 1A and A1). Thus, individual ganglion cells never get enough time to express light intensity in spike counts with sufficient resolution. Moreover, integration over different RFs would be possible only if the cortical trajectory of the relevant signal would be known to the readout circuit ahead of time – a mechanism that does not seem to be feasible.

### Sampling of relative depth information by microsaccades

High resolution depth information depends on fine coordination between the eyes. During fixation, eye movements are well coordinated only during microsaccades, during which the eyes move simultaneously to the same direction and roughly at the same amplitude (Krauskopf et al., 1960; St Cyr and Fender, 1969). Luminance changes scanned by such brief microsaccades evoke responses in the retina, as is evident from responses of cortical “saccade cells” (Snodderly et al., 2001). The binocular disparity of these responses is translated to some depth value in the brain (Poggio, 1995; Qian, 1997). This depth value carries an error in absolute coordinates due to misalignment of the eyes which are continuously drifting along different paths (McKee et al., 1990). However, since this error is common to the entire image, *relative* depth relationships can still be resolved, and with the maximal resolution allowed by stereoptic mechanisms (e.g., Archie and Mel, 2000).

How do relative 2D and relative depth computations interact in the visual system is not yet known. However, the complementary nature of the responses of cortical “position/drift cells” and “saccade cells” (Snodderly et al., 2001), suggests that 2D and depth computations might function in parallel employing a time-sharing mechanism: 2D relationships are computed in a periodic manner as describe above, whereas depth relationships are updated during brief interleaving microsaccades, to which the 2D mechanisms are “blind.” An example to illustrate such a process would be the “cycle stealing” mechanism serving direct memory access in computers: memories are updated directly by a peripheral device during brief episodes to which the central processing unit is blind (see, for example, Horowitz and Hill, 1980).

### Examples of human FeyeM

FeyeM were recorded from the right eye of human subjects at 240 Hz and a resolution of 0.01° by the iView-X system (SensoMotoric Instruments, GMBH). Subjects looked at a computer screen distanced 1 m ahead of them and either tried to keep fixation on a single spot marked with a cross or viewed an image freely. Figure A1 depicts 10 FeyeM traces recorded from one human subject while fixating on a single spot within an otherwise blank screen. Figure A2 depicts (i) 50 epochs of 300 ms each from traces of subjects wearing masks with artificial eyes (Figure A2A) – these traces depict the noise induced by the recording system and by head movements; (ii) 50 epochs of 300 ms each from traces of subjects fixating on a single spot surrounded by horizontal or vertical gratings (Figure A2B); (iii) 50 fixational pauses of subjects while freely viewing a natural image (Figure A2C; note the different time scale) and their power spectra (Figure A2D). Fixational pause was defined as the period starting 30 ms after a saccade and ending 10 ms before the next saccade. The distribution of fixational pauses had a mode around 300 ms; only pauses whose duration was >250 ms are depicted. The 50 epochs were selected in these three cases according to the same criteria, exhibiting the largest modulation depths in each data set, and were de-trended.

### Dependency of *R*_out_ on image velocity

Dependency of *R*_out_ on image velocity is depicted in Figure A3.

## Appendix 3

### Spatial encoding of hyperacuity by firing rates

Excellent vernier alignment is achieved with just two or three dots (e.g., Westheimer, 1987; Carney et al., 1995). The location of each dot can be estimated from the firing rates of all ganglion cells activated by that dot. We are not interested in the exact estimation mechanism here. We ask: for how long should that neuronal population be active before a detectable difference in spike count emerges for a hyperacuity offset. For simplicity we approximate the point spread function to a linear decay. We assume that the center of the activated population is firing at *R* spikes/ms, and this rate decreases to 0 linearly across *N* neurons, where *N* is the total number of ganglion neurons that can potentially participate in this computation.

We express the vernier offset, *d*, in units of cell numbers (*d* < 1); thus, *N* and *d* share the same units. We look at the resolution of a population of *N* neurons: what is the minimal offset of a stimulus that will change the population spike count by one spike during time *T*. The difference (*D*) in population spike count induced by an offset *d* during 1 ms of response is the difference in the area of two triangles, of lengths *N* and *N* − *d*, and heights *R* and *R*(*N* *−* *d*)/*N*. These triangles represent the firing probabilities of the population of neurons in which activity would change in the same direction, e.g., increase, had their stimulating dot moved by *d*.

$$D=0.5\left(RN-\frac{R{\left(N-d\right)}^{2}}{N}\right)=\frac{R}{2N}\left(2Nd-d^{2}\right)\;\left[\text{spikes/ms}\right]$$

Since 2*Nd* > > *d*^2^,

D≈Rd[spikes/ms]

Thus, the difference does not depend at all on *N*, but on the offset and the maximal firing rate in response to such stimuli. This would be true in general for any gradually decreasing function, since as the spread becomes wider, more cells are involved, but at the same time the slope becomes smaller and the difference in activity induced by the two positions decreases. Indeed, numerical solutions of Gaussian-like decaying functions, such as the point- or line-spread functions specified for pupil diameters around 3 mm (Wachtler et al., 1996) show similar independence on *N* for *N* > ∼4, and similar or smaller *D* values.

The time window required for achieving one spike difference in such retinal populations is thus:

$$T\geq \frac{1}{Rd}\left[\text{ms}\right]$$

The maximal retinal response rate observed with line vernier stimuli was *R* = 1/20 spikes/ms (Wachtler et al., 1996). Vernier offsets at threshold are smaller than 1/4 of the smallest cone diameter. For *d* = 1/4 we get *T* ≥ 80 ms. Thus, it would take at least 80 ms to get a difference of 1 spike *across the entire population* of the relevant retinal ganglion cells.

## Abbreviations

FeyeM, fixational eye movements; FF, feedforward; NPLL, neuronal phase-locked loop; PD, phase detector; RCO, rate-controlled oscillator; SC, simple cell; sRF, subfield of a simple receptive field.
